# Annotation of the Extracellular Enveloped Form of Monkeypox Virus for the Design, Screening, Validation, and Simulation of a Chimeric Vaccine Construct

**DOI:** 10.3390/biology14070830

**Published:** 2025-07-08

**Authors:** Mohammad Asrar Izhari, Essa Ajmi Alodeani, Siraj B. Alharthi, Ahmad H. A. Almontasheri, Foton E. Alotaibi, Rakan E. Alotaibi, Wael A. Alghamdi, Osama Abdulaziz, Fahad Alghamdi, Ali Alisaac, Mansoor Alsahag, Ahmed R. A. Gosady

**Affiliations:** 1 Faculty of Applied Medical Sciences, Al-Baha University, Al Baha 65522, Saudi Arabia; 2 Department of Dermatology, Al-Kharj Military Hospital, Al-Kharj 11361, Saudi Arabia; 3 Molecular Genetics Unit, Alhada Armed Forces Hospital, Taif 26792, Saudi Arabia; 4 Ibn Sina Hospital for Extended Care, Makka 24247, Saudi Arabia; 5 Genetic Counselling Department National Guard Hospital Riyadh, Riyadh 11426, Saudi Arabia; 6 College of Medicine, King Saud bin Abdulaziz University for Health Sciences, Riyadh 11481, Saudi Arabia; alotaibi.rakan0143@gmail.com; 7 Laboratory Department, King Faha Hospital, Al-Bhaha 65732, Saudi Arabia; 8 Department of Clinical Laboratory Sciences, College of Applied Medical Sciences, Taif University, Taif 21944, Saudi Arabia; 9 Prince Mishari Bin Saud Hospital, Baljurshi 65639, Saudi Arabia; 10 Laboratory Department, Baish General Hospital, Jazan 87597, Saudi Arabia

**Keywords:** monkeypox, docking, immunity epitope, immunoinformatics, cloning, simulation

## Abstract

Recent outbreaks caused by hMPXV, especially hMPXV lineages/sub-lineages, represent public health threats necessitating stringent prophylactic measures to ameliorate their colossal impact. The current study annotated the EEV form of hMPXV’s target proteins to formulate a reverse vaccinology-dependent hMPXV multiepitope vaccine. Epitope determination, followed by vaccine formulation, was undertaken. The promising formulation was validated for its potential to trigger immune responses immunoinformatically. The MPXV-1-Beta formulation was characterised as a promising candidate based on antigenicity score, physicochemical properties, solubility score, ProSA Z-score, and Ramachandran plot. Docking, normal mode analysis, and molecular dynamic simulation of MPXV-1-Beta with TLRs and MHCs authenticated rigid docking and its efficacy in enhancing immune receptor activation under physiological conditions. MPXV-1-Beta was discerned to trigger a sustained immune response (IR) with a broader average population coverage of 97.526, SD = 12.44. The proposed MPXV-1-Beta candidate showed significant potential. The findings of this study provide a preliminary framework for developing an efficacious hMPXV vaccine; however, extensive in vitro, in vivo, and clinical evaluations are required to substantiate the computational insights.

## Simple Summary

The human monkeypox virus (hMPXV) is a recently growing public health concern, prompting the necessity for effective vaccines. In this study, reverse vaccinology was leveraged to design a multi-epitope vaccine formulation (MPXV-1-Beta) targeting the extracellular enveloped virus (EEV). The safety, efficacy, and capability of the MPXV-1-Beta vaccine to trigger strong immune responses was assessed. The MPXV-1-Beta exhibited high antigenicity, good solubility, and structural reliability. When assessed through docking and simulations, it reflected stable interactions with TLR2, TLR4, and MHC molecules, suggesting its ability to elicit an immune response effectively with over 97% of global population coverage. These results highlight MPXV-1-Beta as a potential vaccine formulation against hMPXV. However, further laboratory and clinical validation is needed.

## 1. Introduction

The hMPXV strain of MPXV is responsible for causing human infection via the respiratory route [1], skin [2], and sexual contact [3]. One interesting fact that surfaced during recent outbreaks is a higher prevalence of hMPXV cases among men who are sexually engaged with other men [4,5]. The hMPXV strain, belonging to the genus Orthopoxvirus, causes zoonotic infection [6], symptomatologically similar but with lower severity [7] compared to variola virus (VARV) [8], which remained endemic in Africa for a long time [9]. However, reports of recent outbreaks beyond the usual endemic region (African content) raised global public health concerns. Moreover, the remarkable surge of African cases with international and intercontinental travelling led to worldwide outbreaks in 2022 [10]. Between January 2022 and October 2023, across one hundred and fifteen countries, approximately 91 thousand confirmed cases were recorded, which led the WHO to declare a worldwide public health emergency [11]. Clade I (virulent and severe) has high mortality, and Clade II (subclade IIa & subclade IIb) has a comparatively lower mortality of hMPXV, which has been reported [12]. Clade IIb, first identified in the non-endemic region (Europe and North America) in 2022, has been recognised as a 2022–2024 pandemic strain with mutations responsible for enhanced human-to-human transmissibility. Further intra-Clade IIb divergence, with evolving lineages/sub-lineages affecting human-to-human transmissibility, symptom severity, and therapeutic effectiveness, would be a major future challenge in curbing the spread of the hMPXV [13].

The definitive treatment for hMPXV does not exist; however, therapy and supportive care measures for smallpox are the only choice for clinical management of hMPXV disease [14], and vaccines (ACAM2000 and JYNNEOS) approved against smallpox are considered protective against hMPXV infection [15,16]. Interestingly, individuals with prior hMPXV infection or those vaccinated against smallpox were reported to be comparatively safer in contracting a fresh infection during recent outbreaks [4]. A possible contributing factor for ongoing outbreaks could be the waning cross-reactivity-based protection offered by smallpox vaccines against hMPXV infection [17,18] due to the emergence of novel hMPXV variants/sub-variants, especially Clade IIb, a global outbreak strain [19]. Additionally, another challenge is the safety issues (myocarditis and pericarditis) associated with the currently available smallpox vaccines, especially in pregnant and eczema patients [20]. Therefore, developing a definitive treatment for hMPXV and a vaccine against hMPXV is noteworthy.

The EEV and intracellular mature virus (IMV) are the well-reported infectious forms of hMPXV [21,22]. The highly conserved central genomic region encompasses the genes that encode hMPXV structural proteins (SP), expressed in different hMPXV forms, which are suitable targets for vaccine design [23]. EEV’A35R is a crucial SP analogous to vaccinia virus (VACV) ‘s A33R, an envelope glycoprotein that facilitates actin-rich microvilli formation to enhance the spread of viral particles between cells [24]. EEV’ B6R is a critical SP analogous to VACV’s A33R, a glycoprotein undergoing palmitoylation crucial for protein trafficking to the membrane and hence IMV-to-EEV wrapping/switching [25,26]. A35R and B6R have been targeted as part of the few polyvalent vaccines developed against hMPXV; however, it is worth characterising potential antigenic determinants for the design and simulation of a vaccine targeting these SPs of the EEV for hMPXV to curb cell-to-cell spread and IMV-to-EEV switching [26].

By leveraging machine learning (ML) algorithm-based computation immunological tools/servers/programs, multiepitope peptide construct development has gained remarkable significance in recent years, especially during the COVID-19 pandemic [27]. Modern mRNA vaccine candidate characterisation leveraging computational algorithms fosters faster vaccine design and computational validation against emerging variants/sub-variants of virulent viruses, including hMPXV. Therefore, in this study, two specific proteins (A35R and B6R) of EEV forms of hMPXV were targeted to annotate the highly potential antigenic determinants (epitopes) to design a chimeric mRNA vaccine formulation and to validate the effectiveness of the construct. Multiepitope vaccine formulations are devised to elicit strong immune responses without incorporating live viruses, which reduces the health risk. The reverse vaccinology approach fosters the identification of precise epitopes and adjuvants, enhancing vaccine efficacy. Moreover, the scalability allows swift vaccine deployment to improve outbreak preparedness. Additionally, the outcome of this study was also the characterisation of a pool of epitopes from A35R and B6R proteins, which could be of therapeutic or diagnostic value.

## 2. Materials and Methods

**Figure 1:**
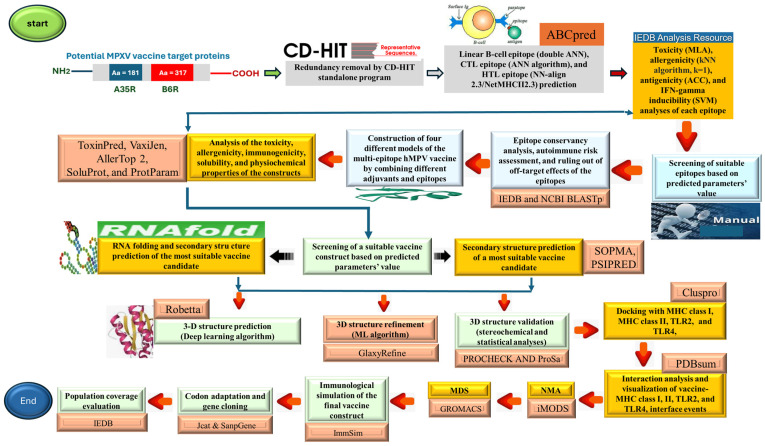
Illustration of the methodological framework. NMA = normal mode analysis, and MDS = molecular dynamic simulation. All the servers/tools depicted in this figure have been described in Section 2.

Figure 1 presents a detailed workflow and methodological foundation, outlining the structured sequence of processes.

### 2.1. Target Proteins, Their Acquisition, Query Dataset Generation, and Antigenicity Determination

Initially, FASTA-formatted sequences of hMPXV/EEV proteins (A35R and B6R) were acquired, duplicates were removed, and two distinct query datasets were created. Two databases, BV-BRC [28] and Uniport [29], were accessed to obtain the required target protein sequences for further analyses. Retrieving sequences from two different databases provides additional advantages to enhance the breadth, completeness, and cross-validation of protein sequence datasets used for downstream analyses. CD-HIT (it uses a greedy incremental clustering algorithm in combination with a short word filtering method to identify the similarities in the sequences), a redundancy-removing standalone tool, was employed to eliminate the possible redundancy of the sequences, utilising a similarity threshold (90%) to recover the non-paralogous sequences and to avoid the bias within each dataset and ensure the accuracy and reliability for large-scale analysis [30] (Figure S1 of Supplementary File S1). Afterwards, NCBI BLASTp was used for screening the selected proteins against human protein sequences with particular parameters (E-value ≥ 10^−4^ and the identity < 50%) to identify the non-paralogous hMPXV-specific EEV protein sequences. Evaluating the antigenicity of the target proteins is paramount in vaccine design. Therefore, acquired protein sequences were assessed for their antigenic potential using the VaxiJen server [23], which provides an antigenicity score. Other significant features of EEV target proteins, such as physicochemical features, the toxicity of proteins, allergenicity status, and the expression solubility of EEV target proteins, were assessed by using Expassy/ProtParam (a protein system analysis server), ToxinPred (toxicity profiling server) [23], AllerTop (allergenicity prediction server) [23], and soluProt (protein solubility determiner), respectively.

### 2.2. Determination of Epitopes

Computational analyses were carried out to predict linear B-cell epitopes (LBEs), critical for generating antibody-mediated immune responses. Immunoinformatics methods were used to identify cytotoxic T-lymphocyte (CTL) antigenic determinants/epitopes. Helper T-lymphocyte (HTL) antigenic determinant/epitope prediction, an integral part of cell-mediated immunity, was conducted using IEDB tools.

#### 2.2.1. Linear B-Cell Epitopes (LBEs)

The targeted sequences of EEV proteins were processed on the ABCpred to identify and characterise LBEs [31]. An ABCpred score cutoff (score = 0.51) was laid down to predict 16-mer LBEs. The function of the immunoinformatic server (ABCpred) is based on a neural network algorithm to compute the specificity, sensitivity, accuracy, and positive predictive score for each immunogenic LBE [31].

#### 2.2.2. CTL Epitopes (CTLEs)

To characterise the CTLEs, computational analysis was carried out by harnessing the IEDB consensus methodological strategy (ANN 4.0), exploiting artificial neural networks (ANNs) heavily trained on the huge data of experimentally obtained binding affinity. ANN 4.0 is meant to enhance its prediction accuracy by operating on large, comprehensive datasets, rendering it considerably effective for CTLE identification [32]. CD8+ T-cell activation is the initial step in cell-mediated IR (CMIR) development. To prevent the unsatisfactory immune activation of CD8+ T-cells, only the CTLEs exhibiting an IC_50_ value smaller than the restraining threshold (Th = 100 nM) were prioritised as candidates of choice for addition to the proposed vaccine construct in the current study.

#### 2.2.3. HTL Epitopes (HTLEs)

The HTLEs, critical components of the vaccines, were computationally characterised using the neural network-based alignment (NN-align 2.3)/ML-dependent-NetMHCII 2.3 methods provided on the IEDB platform. HTLEs with high ranking were manually screened for constructing different models of the vaccines. Interferon-gamma (IFN-γ)-inducing HTLEs [33] and IL-4-inducing HTLEs were ascertained by using IFNepitope, which functions on ML-model and IL-4pred, respectively [34].

### 2.3. Multi-Faceted Assessment of Epitopes (LBEs, CTLEs, and HTLEs) for Screening Epitopes Suitable for Vaccine Design

The appropriateness of the antigenic determinants, such as LBEs, CTLEs, and HTLEs, was ascertained and thoroughly examined for their antigenic feature (VaxiJen 2.0) [35], toxicity profile (ToxinPred) [36], soluble expression (SoluProt 1.0) [37], and allergenicity (Allertop) [38]. In addition, nontoxic, nonallergenic, and soluble LBEs were screened based on the score obtained from ABCpred to incorporate and develop effective constructs. Moreover, the CTLEs and HTLEs exhibiting pertinent characteristics (nontoxic, nonallergenic, and soluble) were ascertained based on their antigenicity scores retrieved from VaxiJen 2.0 analysis to add to the constructs. Moreover, all the predicted epitopes were screened for sequence conservation using the IEDB Conservancy Analysis tool (http://tools.iedb.org/conservancy/, accessed on 27 June 2025), which compares each epitope against a panel of A35R and B6R protein sequences of different hMPXV strains, including Clade I and Clade II, to calculate their percentage identity and distribution. To rule out autoimmune risks, the selected epitope sequences were processed on NCBI’s BLASTp tool (https://blast.ncbi.nlm.nih.gov/Blast.cgi, accessed on 27 June 2025) against the human proteome (taxid: 9606), and to assess possible off-target effects, selected epitopes were further subjected to NCBI’s BLASTp tool against the Vaccinia virus (taxid: 10245) proteome.

### 2.4. Vaccine Model Construction (Formulation) and Screening for Efficient Vaccine Prototypes

Amino acid linkers play a vital role in vaccine design by organising multiple epitopes in a structured manner, improving antigen detection and immune system recognition. Purposefully, the LBEs, CTLEs, and HTLEs were fused using specialised amino acid linkers to produce two (*n* = 02) hMPXV vaccine prototypes, initially. Subsequently, these two initially designed prototypes were conjugated with three kinds of adjuvants, (1) mammalian beta-defensin, (2) heparin-binding hemagglutinin adhesin (Hbha), and (3) ribosomal protein, to generate the six (*n* = 06) different prototypes which were later screened to figure out the most potent prototype among them all. LBEs were connected using GPGPG linkers, HTLEs were fused with KK linkers, and AAY linkers were employed for CTLEs [39]. The antigenic and physicochemical properties of the six design prototypes were assessed for screening purposes for the most potent prototype. To ensure the desired effectiveness and appropriate safety, a vaccine must exhibit stability, non-allergenicity, antigenicity, nontoxicity and high solubility. Thus, the physicochemical attributes, antigenic potential, allergenic profile, toxicity, and expression solubility of all six prototypes were accurately ascertained by leveraging the ProtParam, VaxiJen v2.0 [35], AllerTop [38], ToxinPred, and SoluProt 1.0 [37] servers, respectively. Based on these characteristics, the most suitable and potent vaccine prototype was screened as MPXV-1-Beta out of six designed prototypes (MPXV-1-Hbha, MPXV-1-Beta, MPXV-1-Ribo, MPXV-2-Hbha, MPXV-2-Beta, MPXV-2-Ribo).

In addition to the epitopes, in the vaccine sequences, a non-natural-pan-DR-epitope (a synthetic HTLE engineered to bind efficiently to a broad level of MHC class II for activating HTL) was integrated into the vaccine prototypes to strengthen the potency and efficiency of the vaccine. The Kozak sequence, a conserved nucleotide motif encompassing the start codon (AUG), has been recognised as one of the most critical components (it ensures precise translation initiation and prevents ribosome scanning errors) of the mRNA vaccine. The Kozak sequence was added to the mRNA vaccine sequence in addition to the antigenic determinants. Appropriate adjuvants and functional linkers were also added to get optimum translational efficiency [40]. Another element, a stop codon (UGA), was enhanced after the antigen coding sequence, for efficient termination with minimal readthrough [41]. A signal peptide sequence essential for the extracellular secretion of translated epitopes and an MHC-class-I-trafficking-signal (MITD) linked to the terminus (C-terminus) of the antigenic protein represent two crucial elements for the optimum efficiency of the construct. MITD is needed to ensure antigen presentation via the MHC-I Pathway. Therefore, the 5′ region of the open reading frame (ORF) was framed to include the tissue plasminogen-activator (tPA) secretory signal sequence retrieved from UniProt (UniProt/ID-P00750) at the N-terminus of the antigen coding sequence, while the MITD sequence retrieved from UniProt (UniProt/ID-Q8WV92) was added at the C-terminus of the antigen-coding sequence in the 3′ regions of ORF [42,43]. mRNA is naturally unstable because of its single-stranded nature, impacting its lifespan, protein synthesis efficiency, and immune response, which creates additional challenges in vaccine design. To address the issue, components typically found in eukaryotic mRNAs must be incorporated into the vaccine construct [44,45]. Therefore, to counteract the mRNA instability issue, the vaccine prototype was engineered by integrating four well-known significant components (5′ m7G cap, poly(A) tail, 5′ untranslated regions (UTRs) for ribosome recruitment, and 3′ UTRs to mitigate degradation). These critical elements were integrated to improve mRNA stability and translational efficiency [46]. Poly (A) tail engineering necessitates standardisation of the segment length of the poly(A) tail because excessively short or overly long tails affect protein translation efficiency [47]. An optimal poly(A) tail length of approximately 115–150 nucleotides is optimal for the desirable mRNA vaccine efficacy [48]. Moreover, to achieve the desired results, two other significant components were incorporated; NCA-7d was integrated into the 5′ UTR to stabilise the mRNA structure, while S27a + R3U was added into the 3′ UTR to prolong mRNA half-life and upgrade translation efficiency [49,50].

### 2.5. Secondary-Level Structural Analysis of the Vaccine

The secondary structural composition of the selected vaccine prototype (MPXV-1-Beta), including beta-sheets, coils, and alpha-helices, was computationally predicted using SOPMA [51] and the PSIPRED 4.0 servers [52]. These servers ascertain key structural components, such as transmembrane helices, topology, and folding patterns within a specific peptide sequence, to give comprehension of the stability and functional features of the vaccine MPXV-1-Beta. The mRNA secondary structural feature analysis was accomplished by employing the RNAfold of ViennaRNA (v. 2.0) [53], which carves the centroid secondary mRNA structure and determines McCaskill’s algorithm-based estimation of the pertinent metrics, like minimal free energy (MFE) [54].

### 2.6. The Three-Dimensional Structure of MPXV-1-Beta and Its Computational Refinement

Analysing the 3D structure of a vaccine construct plays a key role in assessing its stability, functionality, and immune response potential. The 3D structural information supports improvements in vaccine formulation, immune response (IR) potential, and overall effectiveness. The 3-dimensional structure of the most potent vaccine prototype (MPXV-1-Beta) was modelled by leveraging the Robetta server, ensuring accurate and reliable structural insights to evaluate the vaccine constructs’ stability, folding, and functional capability [55]. The 3D structure underwent further refinement through Galaxy Refine, utilising the pdb file generated by the Robetta server [56]. Upon refining and enhancing the structural properties, the PROCHECK server was employed to optimise the structure quality of the model. Moreover, the prototype underwent validation of the quality of the structure through the ProSA server [57].

### 2.7. Docking and Normal Mode Analyses of MPXV-1-Beta Vaccine for Gaining Analytical Insight into Molecular Interactions

A vaccine must form precise and stable conformational interactions with primary immune receptors to initiate a lasting, robust, and specific immune defence. The pdb-format files corresponding to Toll-like receptors TLR4 (PDB ID: 4G8A) and TLR2 (PDB ID: 2Z7X) were sourced from the Protein Data Bank (PDB). Additionally, the files (in pdb format) of MHC class I (PDB ID: 2XPG) and MHC class II (PDB ID: 1KG0) were also retrieved from the same PDB database. Harnessing Pymol software (2.5.0), preprocessing (manipulation of the chain identifier) was performed on these TLR and MHC pdb files to make them suitable for further analysis. Subsequent to the preprocessing of the pdb files, ClusPro 2.0-based receptor docking with the MPXV-1-Beta prototype was executed [58]. Afterwards, PDBsum (web-based bioinformatics tool/server), in conjunction with the application of Pymol script/commands, was leveraged to evaluate and visualise interaction events (at residues and atomic level) at the interface of every complex [59]. The iMODS server, which provides platforms for normal mode analyses (NMAs) of biomolecular structures, primarily protein complexes, was leveraged to understand the vaccine–receptor docked complexes’ stability. iMODS was employed to perform the NMA to determine the intrinsic dynamic behaviour and flexibility of the molecular structure of the immune molecule–vaccine formulation complexes, to gain further insights into molecular motions and flexibility by evaluating the intrinsic vibrational modes of the complexes [60].

### 2.8. GROMACS-Based Molecular Dynamics Simulation of the TLR-Receptor (TLR-4 and TLR-2)-MPXV-1-Beta Complexes

MDS for the TLRs-MPXV-1-Beta formulation was performed using the GROMACS platform for 100 ns to assess the stability of receptor–vaccine complexes in an aqueous solution to comprehend the binding interactions of receptors with MPXV-1-Beta [60]. The system was prepared and parameterised using the AMBER99SB force field. The complexes were solvated in a triclinic simulation box (1.0 nm padding between the solute and the box boundary to prevent interactions with its periodic images under periodic boundary conditions (PBCs); this is a critical step to ensure appropriate solvation and proper electrostatic calculations and to minimise artefacts in non-bonded interactions) [60,61] filled with explicit water molecules modelled by the SPC (Simple Point Charge) water model (a commonly used model in protein simulations for its computational efficiency and accuracy) [61] to mimic the aqueous environment, followed by neutralisation by physiological-concentration Na^+^ or Cl^−^ ions (concentration of NaCl neutraliser = 0.15 M or 150 mM) to maintain the physiological ionic strength and charge or electrostatic neutrality [62]. Energy minimisation was executed by employing EM-integrator (steepest descent algorithm) to relax the initial configuration of the complexes with EM steps of 5000. The whole system was equilibrated to achieve thermodynamic equilibrium at 310 K and 1 bar pressure for an equilibration time (100 ps) under NVT, followed by NPT ensembles. Temperature coupling (to maintain constant temperature) and pressure coupling (to allow the volume of the simulation under the target pressure to adjust thermodynamically) were applied. Finally, after the equilibration process, production MDS was executed for a total duration of 100 nanoseconds (ns) with the leap-frog simulation integrator, and the total number of frames saved was 5000 [60]. A post-simulation study was carried out to assess the RMSD (root mean square deviation), RMSF (root mean square fluctuation), the number of hydrogen bonds, and Rg (gyration radius) on the MDS trajectory obtained through Gromacs. Moreover, the Molecular Mechanics Poisson–Boltzmann Surface Area (MMPBSA) method was used to estimate the binding free energy (ΔG bind) for the last 20 ns of the simulation [61].

### 2.9. Codon-Adaption Execution and Vector-Based Cloning of MPXV-1-Beta Formulation

MPXV-1-Beta’s sequence was subjected to the web-based server for reverse translation. The DNA sequence of MPXV-1-Beta acquired after reverse translation was processed for codon adaptation, exploiting the Java-Codon-Adaptation Tool (JCAT) algorithm to boost and determine the construct’s translational yield and expression capability [62]. The GC content (in the DNA sequence) and codon adaptation index (CAI) were ascertained. A CAI of 1.0 was laid down as an ideal cutoff value, while scores better than 0.8 were considered optimal for robust and sustained expression. The GC content (most desirable range = 30% to 70%) was also determined, as deviations beyond this threshold may negatively affect transcription and translation processes, thus impacting protein expression levels [63]. pET28a (+), an expression vector, was selected, and the *E. coli* strain, as an expression system, was sourced from the SnapGene tool to perform cloning of the meticulously optimised (by JCAT) MPXV-1-Beta vaccine sequence [64].

### 2.10. Simulation for Evaluating IR

MPXV-1-Beta’s immunological potential was assessed through computational approaches harnessing the C-ImmSim server/tool. The C-ImmSim server operates at a cellular level, and it is an agent-dependent informatics algorithm that precisely simulates the intricate interactions within the mammalian defence system. The server signifies the detailed features of the IRs triggered by antigens (MPXV-1-Beta). The server systematically determines the IRs to the proposed vaccine MPXV-1-Beta [65]. The simulation was conducted for the initial dose (at 0 days) followed by a couple of booster doses (first booster at 30 days and second booster dose at 60 days). All parameters, encompassing random speed and MHC class I and II alleles, were set as server default parameters. The parameters were configured with a volume setting of ten, and the impact was evaluated for 300 days.

### 2.11. Population Coverage

Finally, the population coverage was determined by taking into account all the alleles of both classes of MHCs by harnessing the IEDB analytical resource [66]. Based on HLA allele distribution, the population coverage analysis resource available with IEDB computes the percentage (%age) of individuals within a population that may respond to a provided group of epitopes (CTLEs and HTLEs). The population coverage analytical resource, which performs at standard parameters (default parameters), was harnessed to perform the analysis.

## 3. Results

### 3.1. Eligibility Assessment of the Target Proteins

Two EEV target proteins’ sequences, A35R (*n* = 575) and B6R (*n* = 1191), were processed (retrieved on 6 November 2024). The accession numbers of the two prioritised virus-specific non-paralogous protein sequences for the downstream evaluation were A35R (an: QJQ40286.1) and B6R (an: AAL40625.1) (Figure S1 of Supplementary File S1). These two EEV proteins were processed to predict appropriate LBEs, CTLEs, and HTLEs. Furthermore, the physicochemical characteristic assessment of the target proteins revealed the hydrophilic profile of the A35R and B6R proteins (Grand Average of Hydropathicity (GRAVY) cutoff < zero): A35R/EEV (GRAVY = −0.305) and B6R/EEV (GRAVY = −0.182). Two (*n* = 02) target proteins were identified as nontoxic and nonallergenic, which is vital for a protein to be a suitable candidate (Table 1). The antigenicity score (A35R/EEV = 0.4976 and B6R/EEV = 0.5786), solubility, AA chain length, theoretical pI (TpI), and other physicochemical parameters of the two proteins are mentioned in Table 1.

### 3.2. LBE Determination

LBEs, a vital component for a vaccine formulation to be effective, were determined. The most appropriate and promising LBEs were chosen by considering their antigenicity score, which demonstrates their strong interaction with B-cell receptors (BCRs), which is an essential factor in activating humoral (Ab-mediated) IR. Multi-faceted criteria for screening LBEs were adopted, for example, high binding ABCpred scores, antigenicity (beyond a threshold/cutoff of 0.4), nontoxic nature, and nonallergenicity. Six (*n* = 06) LBEs (*n* = 03 from each EEv protein) were evaluated for incorporation into the final formulation of the vaccine. The binding affinity based on the ABCpred scores, antigenicity scores, toxicity profile, and allergenicity of the LBEs are summarised (Table 2).

### 3.3. CTLEs Determination

CTLEs were assessed because CTLs/CD8+ T-cells are pivotal in defending against viral infections. The CTLEs with IC_50_ values < 100 nm were prioritised for further evaluation. The CTLEs with nontoxicity, nonallergenicity, and positive antigenicity values (top-ranked; score > 0.5) were carefully chosen to incorporate into the vaccine construct. Six (*n* = 06) CTLEs (*n* = 03 from each target EEV protein) were picked for integration into the final formulation of the vaccine. The CTLEs and their allele, IC_50_ value for each epitope, antigenicity scores, toxicity profile, and allergenicity are mentioned in Table 2.

### 3.4. Determination of Potential HTLEs

Next, 15-mer HTLEs were assessed along with their MHC class II allele pairs by leveraging the MHC-II binding assessment tool (sourced from the IEDB web). HTLEs (IC_50_ value < 100 nm) were selected for formulating the vaccine. Nontoxic, nonallergenic, IL-4-inducing HTLEs with greater antigenicity values (score over 0.5) were chosen for incorporation into the vaccine formulation. Six (*n* = 06) HTLEs (*n* = 02 recovered from individual EEV protein) were picked for integration into the vaccine formulation. On top of that, one (*n* = 01) IFN-γ-inducing HTLEs from A35R/EEV with high scores (IFN-γ scores = 1.267 and AgScore = 0.6529) were screened for inclusion in the vaccine formulation. Table 3 demonstrates the HTLE allele pair, IC_50_ value, antigenicity scores, IL-4pred scores, IL-4-inducing and IFN-γ-inducing potential, toxicity, and allergenicity status of all seven HTLEs.

### 3.5. Epitope Conservancy, Autoimmune Risk, and Off-Target Effect Analyses

A set of *n* = 19 epitopes predicted from the A35R and B6R proteins of different strains/clades of hMPXV were analysed for conservation. Out of 19, 10 epitopes were found to be fully conserved, showing 100% identity across all 30 examined sequences. The identity scores for these epitopes ranged from 93.75% to 100% (Table 4), indicating high evolutionary stability across viral strains/clades. The remaining epitopes exhibited slightly lower but still remarkable conservation (approximately 87–90%). The consistently high conservation rates across most epitopes underscore their potential utility in designing vaccines that aim for broad cross-strain protection against hMPXV.

Furthermore, epitopes were processed on NCBI’s BLASTp against the human proteome. The BLAST results did not show any significant similarity, highlighting the absence of homology to the human proteome. The results indicated that the epitopes were unlikely to elicit cross-reactive immune responses to human proteins, supporting the safety profile of the epitopes for vaccine development.

Moreover, the BLASTp analysis of hMPXV predicted epitopes against the Vaccinia virus proteome revealed moderate sequence similarity (ranging between 58.93% and 66.67%) and partial query coverage (ranging between 44% and 80%). Most of the hits were to EEV membrane glycoproteins, B5R proteins, and plaque-size/B5 glycoprotein (OPG190) in Orthopoxviruses, as some low-to-moderate similarity was expected. However, despite statistically significant matches (low E-value: 5 × 10^−14^), none of the epitopes shared high identity (≥80%) or full-length alignment with Vaccinia or related virus proteomes, which indicates that the epitope specificity to hMPXV has minimal possibility of cross-reactivity to the Vaccinia virus. Therefore, it reduces the concerns related to off-target IR or pre-existing Orthopoxvirus immunity.

### 3.6. Vaccine Formulation and Screening for Potential Vaccine Formulation (Construct)

Two (*n* = 02) vaccine formulations were generated following the integration (in two different combinations) of the evaluated antigenic determinants/epitopes (LBEs = 06 CTLEs = 06, and HTLEs = 06) in addition to one (*n* = 01) IFN-γ-inducing epitope from the A35R/EEV protein using specific linkers for each epitope type. The former two constructs were subsequently combined with three different adjuvants (immune potentiator)—heparin-binding hemagglutinin (Hbha), mammalian-beta-defensin, and ribosomal protein—resulting in the design of six (*n* = 06) vaccine formulations (MPXV-1-Beta, MPXV-2-Beta, MPXV-1-Hbha, MPXV-1-Ribos, MPXV-2-Hbha, MPXV-2-Ribos), which underwent comprehensive analyses. The adjuvants were combined via a linker (EAAAK) to the terminal (N-terminal) of the two previously designed combinations of the vaccine formulation sequences. The amino acid length/molecular weight/TpI of MPXV-1-Beta, MPXV-2-Beta, MPXV-1-Hbha, MPXV-1-Ribos, MPXV-2-Hbha, and MPXV-2-Ribos were 312/32,513.79/9.76, 427/44,894.99/6.13, 426/44,981.24/8.7, 397/40,793.07/8.77, 313/32,427.54/9.48, and 398/40,706.83/6.33, accordingly (Table 5). All six (*n* = 06) formulations were assessed to be antigenic, nontoxic, and nonallergenic. However, the antigenicity score (0.7953) for the formulation MPXV-1-Beta was the maximum, followed by MPXV-2-Hbha (0.7596). Moreover, the expression solubility (Soluproscore = 0.883, cutoff ≥ 0.5) for MPXV-2-Hbha was the highest, followed by MPXV-1-Beta (Soluproscore = 0.83, cutoff ≥ 0.5), as summarised in Table 5. The antigenicity score of MPXV-1-Beta was higher than MPXV-2-Hbha; however, expression solubility (Soluproscore = 0.883) for MPXV-2-Hbha was slightly higher than MPXV-1-Beta (Soluproscore = 0.83). Therefore, the MPXV-1-Beta vaccine formulation was screened as the most promising prototype for downstream analysis. The aliphatic index (AI), GRAVY, and instability index (I-i) of MPXV-1-Beta were assessed to be 74.23, −0.187, and 30.62, respectively. A negative GRAVY score (cutoff = 0) for the selected MPXV-1-Beta exhibited that the formulation was potentially hydrophilic and comparatively appropriately soluble. The I-i score of 30.62 of MPXV-1-Beta indicated that the formulation MPXV-1-Beta was stable and highly soluble. Moreover, the aliphatic index (74.23) between 40 and 85 of the chosen MPXV-1-Beta formulation demonstrated its desirable thermostability and solubility. The physicochemical parameters, antigenicity scores, toxicity status, solubility scores, half-life period, and allergenicity profile for all six (*n* = 06) formulations are outlined in Table 5.

### 3.7. Secondary Structure of MPXV-1-Beta

**Figure 2:**
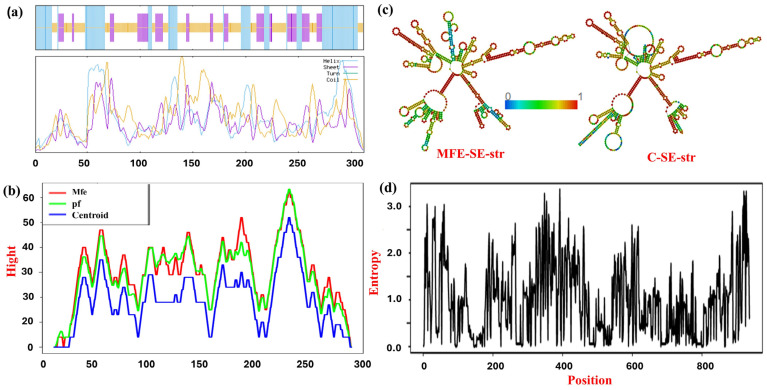
Secondary structure of protein and mRNA of MPXV-1-Beta: (**a**) illustration of the helix, sheet, turns, and coils in the secondary structure of MPXV-1-Beta; (**b**) mountain plot illustrating the mean free energy, thermodynamic ensemble, and centroid structure of mRNA; (**c**) mRNA secondary structure (MFE-SE-str—mean free energy secondary structure; c-SE-str—centroid secondary structure); and (**d**) entropy plot depicting positional entropy for individual position in mRNA. MFE (blue)—minimum free energy structure, showing the most thermodynamically stable configuration. PF (green)—partition function, representing the statistical ensemble of structures contributing to RNA stability; and the centroid (red). The centroid structure is the most representative structure from the ensemble.

The secondary structure of MPXV-1-Beta comprised α-helices (33.65%), 22.12% extended strands, and 44.23% random coils, as demonstrated in Figure 2a and in Figure S2 of Supplementary File S1. The secondary mRNA structure was characterised to have thermodynamic stability, negative minimum free energy (MFE) of the thermodynamic ensemble (TDE) (−358.62 kcal/mol), and a moderate value of ensemble diversity (211.06), which suggests that the RNA structure can adopt a wide range of conformations (representing the flexibility of RNA in folding, which is crucial for its function, such as binding). The centroid secondary structure, demonstrating the ensemble’s most probable or representative structure, exhibited an MFE of −288.1 kcal/mol, as highlighted by dot–bracket annotation (Figure S3 of Supplementary File S1). The structure was slightly less stable (−342.7 kcal/mol) than the global MFE structure; the structure was thermodynamically favourable and represented the RNA’s appropriate conformation. Figure 2c exhibited a few regions with high entropy, whereas other regions represented in blue to green colouration signify lower entropy and greater stability. Most regions exhibited overlapping lines in the mountain plot, which means a sound agreement between the MFE, partition function (PF), and centroid predictions, suggesting a stable and distinct structure in those regions (Figure 2b). Measuring entropy is paramount for comprehending RNA secondary structure; lower entropy signifies positions in the RNA sequence where the predicted structure demonstrates a minimum level of variability. The entropy graph (with positional entropy < 2.0 at most positions) signifies RNA secondary structures’ stability. Nonetheless, the entropy was more than 2.0 at a few positions, which explains the higher flexibility and lesser stability in that particular region (Figure 2d). Overall prediction results suggest the stable RNA secondary structure of the vaccine formulation MPXV-1-Beta.

### 3.8. 3D Structural Evaluation of MPXV-1-Beta

**Figure 3:**
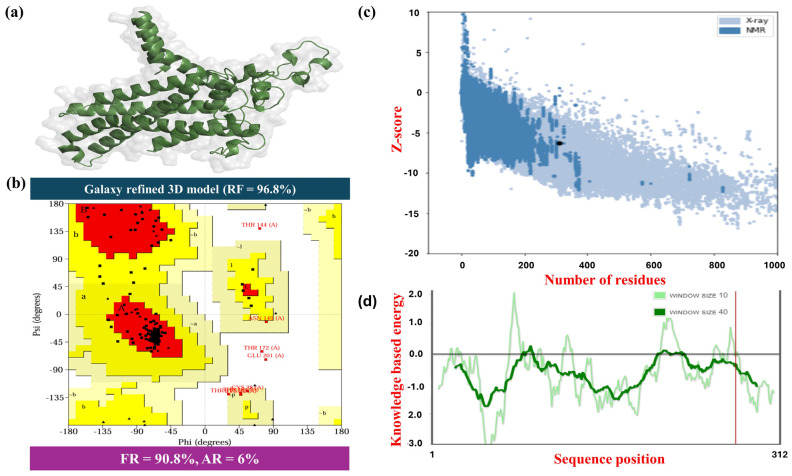
The illustration of the three-dimensional structure of the screened MPXV-1-Beta formulation and its validation: (**a**) Robetta-predicted and Galaxy-refined 3D model of the MPXV-1-Beta formulation, (**b**) Ramachandran plot, (**c**) Prosa-web validation chart, and (**d**) energy validation graph of a 3D model of the MPXV-1-Beta formulation. RF = Ramachandran favoured; FR = favoured region; and AR = allowed region.

The top-ranked GlaxyRefine model of the vaccine formulation, MPXV-1-Beta (Rama favoured 96.8%), was visualised using Pymol and illustrated (Figure 3a). The estimated ProSA (Z-score) was approximately −6.31 for the 3D mode of MPXV-1-Beta, signifying its structural stability and reliability. Additionally, the position of the MPXV-1-Beta formulation in the centre of the graph (black dots) highlights the model’s quality, similar to that of reliable experimentally validated structures (Figure 3b). The authentication of the 3D structure was accomplished by evaluating the backbone dihedral angles in amino acid residues. The two most crucial angles, Phi (Φ) and psi (Ψ), were assessed. The Ramachandran plot, a significant tool for structural validation, demonstrated that 98.8% of all the residues, comprising 90.8% in most favoured regions (MFRs), plus 6.0% in additional allowed regions (AARs) and 2.0% in generously allowed regions, signified a high-quality 3D model (Figure 3c,d). Nonetheless, a few residues were occupied in the disallowed areas (1.2%) (probably due to the flexible loops or spatial conformations), which is predictable and acceptable within limits. Additionally, the stability of the structure’s validation by knowledge-based energy as a function of sequence position was accomplished (Figure 3d). The plots (Figure 3d) showed that sequences had negative energy scores at most positions, demonstrating that the structure was stable in regions showing strong negative peaks. The overall structural validation results substantiated the considerable stability of the MPXV-1-Beta structure for docking analysis of the formulation with appropriate receptors.

### 3.9. Docking of MPXV-1-Beta Formulation with TLRs and Major Histocompatibility Complex Molecules (MHC Molecules) for Interaction Evaluation

**Figure 4:**
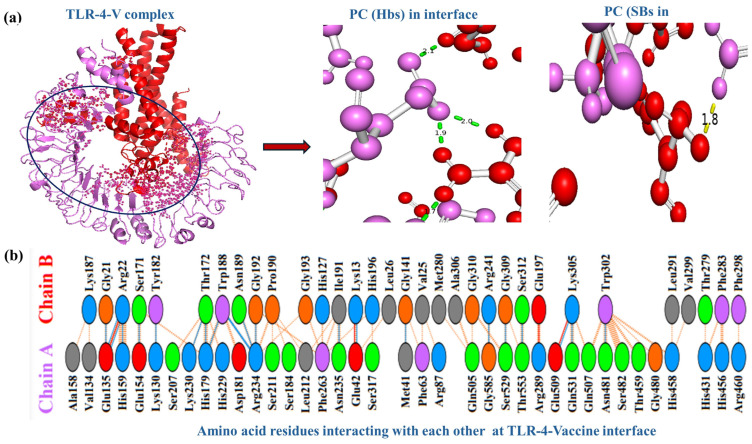
Depiction of MPXV-1-Beta formulation interaction with receptors. (**a**) TLR-4-MPXV-1-Beta interactions (violet colour depicts TLR4 chain, red colour exhibits the MPXV-1-Beta formulation chain, black circular ring on TLR-4-V complex represents interface). PC, polar contacts; Hbs, hydrogen bonds; SBs, salt bridges. (**b**) Illustration of the amino acid (AA) residues interacting in the TLR-4-MPXV-1-Beta interface (Chain A: TLR-4 and Chain B: MPXV-1-Beta formulation).

The MPXV-1-Beta formulation was meticulously docked with TLR4 and TLR2 receptors to comprehend the receptor binding dynamics of the complex. Figure 4a exhibits the molecular interaction interface (where atoms of the MPXV-1-Beta formulation and receptors are in contact) and interface events (at the residue and atomic level) of the TLR-4-MPXV-1-Beta complex, with the formation of the polar contact in the interface (hydrogen bonds plus salt bridges with their computed bond lengths). Figure 4b shows the kinds of amino acid residues of the TLR-4 receptor interacting with the residues of MPXV-1-Beta formulation. There were 38 residues of TL4 receptor and 32 residues of MPXV-1-Beta formulation interacting, involving the formation of *n* = 04 salt bridges, *n* = 22 hydrogen bonds, and *n* = 198 non-bounded contacts with a total interface of 1627–1792 Å^2^, which signifies rigid docking interactions at the interface (Table 5).

**Figure 5:**
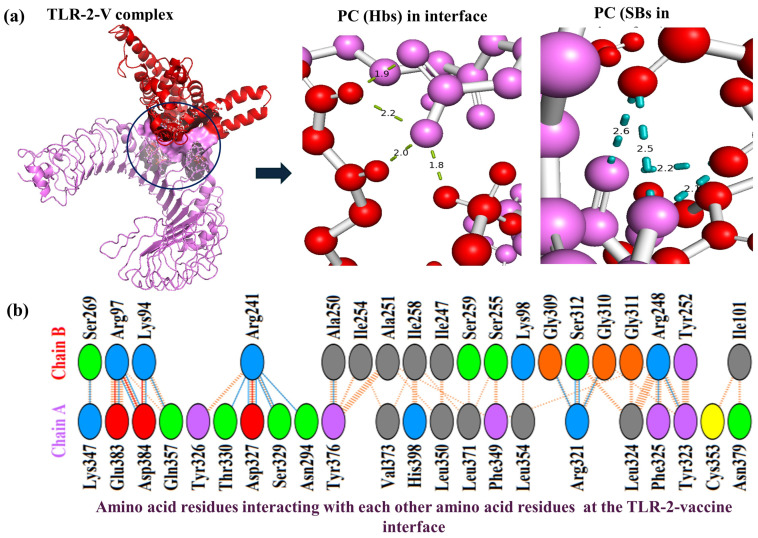
Exhibition of TLR-2-MPXV-1-Beta formulation interaction. (**a**) TLR-2-MPXV-1-Beta interactions (violet colour represents TLR2 receptor chain, red colour exhibits MPXV-1-Beta formulation chain, and black circle represents interface). PC = polar contacts, Hbs = hydrogen bonds, and SBs = salt bridges. (**b**) Demonstration of the amino acid (AA) residues interacting in the TLR-2-MPXV-1-Beta interface (Chain A = TLR-2 receptor and Chain B = MPXV-1-Beta vaccine formulation).

Figure 5a depicts the interaction interface and interfacial events (at the atomic level) of the TLR-2-MPXV-1-Beta formulation complex with the formation of polar contact at the interface (hydrogen bonds and salt bridges along with their computed bond lengths). Figure 5b depicts the kinds of amino acid residues of the TLR-2 receptor interacting with the amino acid residues of the MPXV-1-Beta vaccine formulation. There were 22 residues of TL2 and 19 residues of MPXV-1-Beta formulation interacting, involving salt bridge (*n* = 04), hydrogen bonds (*n* = 19), and *n* = 135 non-bounded contacts with a total interface of 1050–1125 Å^2^, signifying stable docking interaction between the components of the complex (Table 6).

**Figure 6:**
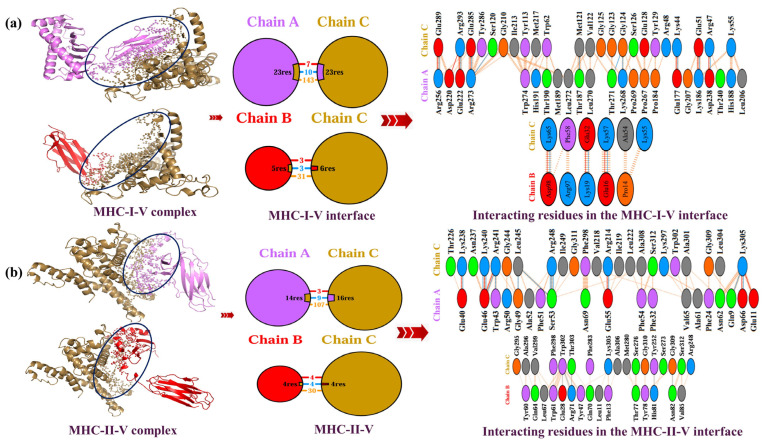
Exhibition of MHC-MPXV-1-Beta interaction. (**a**) MHC class-1-MPXV-1-Beta interactions (sand-colour chain C represents MPXV-1-Beta, violet-colour chain A denotes chain A of MHC class-I, red-colour chain B denotes chain B of MHC class-I, and black circle represents interface between MHC class-I and MPXV-1-Beta). (**b**): MHC class-II-MPXV-1-Beta interactions (sand-colour chain C represents MPXV-1-Beta, violet-colour chain A denotes chain A of MHC class-II molecule, red-colour chain B denotes chain B of MHC class-II, and black circular ring represents an interface between MHC class-II and MPXV-1-Beta).

Docking MHCs with the proposed MPXV-1-Beta formulation is paramount for determining its immunogenic potential. Figure 6a exhibits the interaction between MHC I and the MPXV-1-Beta formulation (MHC I-MPXV-1-Beta interface, number of AA residues of MPXV-1-Beta and MHC I involved in the interaction at the interface, and number of polar and nonpolar contacts along with the types of amino acid residues involved). Figure 6b exhibits the interaction between MHC class II and the MPXV-1-Beta formulation (MHC II-MPXV-1-Beta interface, number of residues of MPXV-1-Beta and MHC II involved in the interaction at the interface, and number of polar and nonpolar bond contacts along with the kinds of amino acid residues involved). A total (chain A plus chain B) number (*n* = 28) of MHC class I residues and *n* = 29 residues of MPXV-1-Beta formulation interacted, involving *n* = 10 salt bridges, *n* = 13 hydrogen bonds, and *n* = 73 non-bounded contacts (Table 6). A total (chain A plus chain B) number (*n* = 34) of MHC class II residues and *n* = 40 residues of MPXV-1-Beta formulation interacted, involving *n* = 06 salt bridges, *n* = 18 hydrogen bonds, and *n* = 251 non-bounded contacts (Table 5). These findings revealed a stable and rigid binding interaction of the MPXV-1-Beta formulation with MHC molecules.

### 3.10. NMA of MPXV-1-Beta Formulation with TLR-Receptors and MHC Molecules

**Figure 7:**
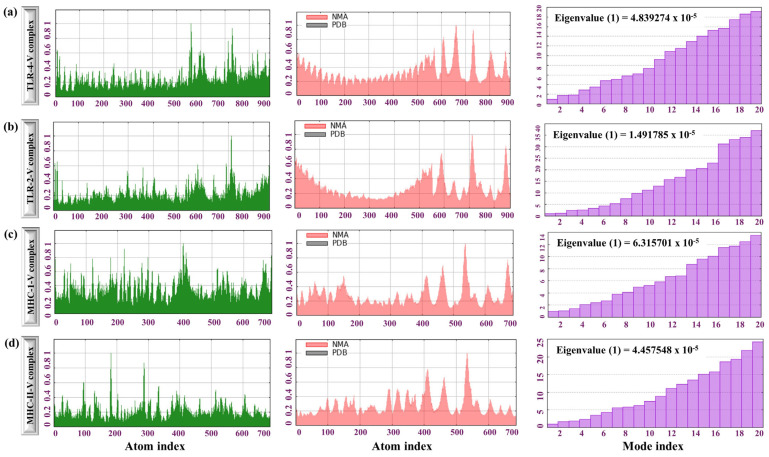
Illustration of the NMA of TLRs-MPXV-1-Beta and MHCs-MPXV-1-Beta. (**a**) NMA results of TLR4-MPXV-1-Beta, (**b**) NMA results of TLR2-MPXV-1-Beta, (**c**) NMA results of MHC class I-MPXV-1-Beta, and (**d**) NMA results of MHC class II-MPXV-1-Beta complexes. Left-side graph: Beta factor/mobility graph (flexibility/deformity value vs. atomic index); middle plot: comparison between the NMA and PDB-derived flexibility data for a protein or molecular complex. The overlay of NMA (pink) and PDB (black) provides detailed insight into the consistency between computational/predicted and experimentally derived structural flexibility and right-side plot eigenvalues computed from the NMA graph against the mode indices.

The dynamic behaviour of the complex TLR4-MPXV-1Beta, TLR2-MPXV-1-Beta, MHC-I-MPXV-1-Beta, and MHC-II-MPXV-1-Beta was analysed to comprehend the flexibility and stability of interactions and to determine the binding effectiveness. The principal deformation plot for the TLR4-MPXV-1-Beta formulation complex revealed that the conformation was stable (most regions showed low flexibility) except for a few long peaks at some positions, highlighting the regions of higher flexibility, which signifies functional or dynamic regions (binding sites, loops, or hinges). The flexible region may be vital for dynamic interactions, such as ligand binding or necessary conformational changes. Additionally, a very low relative eigenvalue (0.48392 × 10^−4^) for TLR4-MPXV-1-Beta in the lower mode (high flexibility) and an increased eigenvalue in the higher mode (increase in stiffness) was observed, indicating a balance between flexibility and stability, which is crucial for biological activity and structural integrity. The low eigenvalue (negative) in the early mode suggests that the structure allows for considerable collective molecular motions necessary for functional flexibility (binding interactions/conformational changes) (Figure 7a). Conversely, the higher relative eigenvalue in higher modes signifies localised structural rigidity, which could correspond to stable structural regions (Figure 7a). Likewise, the relative eigenvalues of TLR2-MPXV-1-Beta, MH C class I-MPXV-1-Beta, and MHC class II-MPXV-1-Beta were recorded to be (0.149178 × 10^−4^), (0.63157 × 10^−4^), and (0.44575 × 10^−4^), as shown in Figure 7b–d, which indicates the appropriate structural stability of the molecular complexes.

Additionally, the deformity graph for TLR2-MPXV-1-Beta, MH C class I-MPXV-1-Beta, and MHC class II-MPXV-1-Beta showed that the structure was stable and rigid, except for a few localised regions with higher flexibility (Figure 7b–d). Overall, the NMA results of the TLRs-MPXV-1-Beta complexes showed their ability to induce robust immune signalling through enhancing immune receptor activation. The NMA results for the complexes (MHC-class-I-MPXV-1-Beta and MHC-class-II-MPXV-1-Beta) signify their stability, reflecting their respective roles in inducing adaptive immunity.

### 3.11. MDS of TLRs-MPXV-1-Beta Vaccine

**Figure 8:**
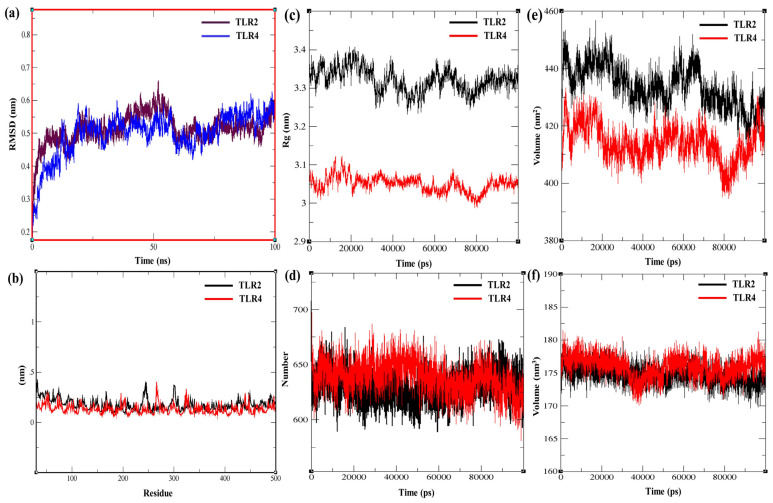
Depiction of RMSD, RMSF, radius of gyration, total SAS area, and SAS volume of TLRs-MPXV-1-Beta vaccine for 100 ns molecular dynamic simulation. SAS = solvent accessible area (in nm^−2^), SAS volume (in nm^−3^), RMSD = root mean square deviation, and RMSF = root mean square fluctuation. (**a**) RMSDs of TLR2/TLR4–vaccine complex, (**b**) RMAFs of TLR2/TLR4–vaccine complex, (**c**) radius of gyration of TLR2/TLR4–vaccine complex, (**d**) number of hydrogen bonds in TLR2/TLR4–vaccine complex, (**e**) total solvent accessible (SAS) area of TLR2/TLR4–vaccine complex, and (**f**) solvent accessible volume of TLR2/TLR4–vaccine complex.

RMSD indicated that both the TLR2-MPXV-1-Beta and TLR4-MPXV-1-Beta complexes achieved equilibrium within the first ~10 ns (vaccine with TLR2 stabilises around 0.5–0.6 nm, while the one with TLR4 does so around 0.6–0.7 nm), as depicted in Figure 8a, which suggested a stable TLR–vaccine complex with no unfolding and major deviation. Per-residue fluctuations (RMSF) were observed to be low for both TLR–vaccine complexes, with minor peaks. The vaccine complexed with TLR2 exhibited slightly higher flexibility at specific residue regions (Figure 8b), indicating that both complexes had a rigid backbone with localised flexibility in loops or terminals. TLR2-MPXV-1-Beta showed a higher Rg (~3.3–3.4 nm) than TLR4-MPXV-1-Beta (~3.0–3.1 nm), indicating that the vaccine complex with TLR2 was relatively more open or extended in conformation compared to TLR4-MPXV-1-Beta, which remained more compact during the simulation (Figure 8c). Rg values were stable over time (100 ns) for both complexes, staying within a ±0.05 nm range (Figure 8c), which is suggestive of no significant fluctuation in compaction and expansion, reflecting the compactness and integrity of the complexes. Stable H-bond counts (600–680) supported stable and cohesive intermolecular interactions, indicators of structural integrity, and binding affinity (Figure 8d). The total SAS area (nm^2^) for both TLR2/TLR4-MPXV-1-Beta complexes showed visible downward trends (stable and steady decrease) over the simulation time, which indicates increasing tightness of the interaction of vaccine with receptors (compactness and stabilisation) and decreasing interactions with the solvent (Figure 8e). A slight fluctuation (175–180 nm^3^) in SAS volume was observed without major trajectory deviation, which suggests stable hydration and equilibrium of the system during the 100 ns simulation (Figure 8f).

**Figure 9:**
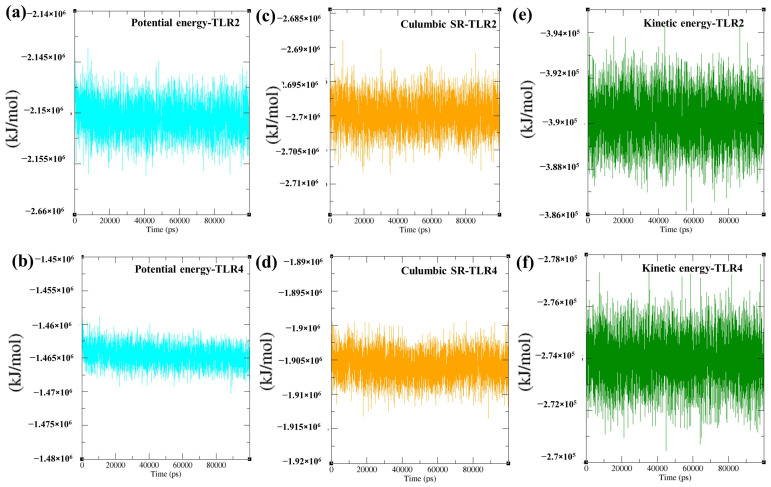
Depiction of total potential energy (in KJ/mol), Coulombic SR (in KJ/mol), and kinetic energy of TLRs-MPXV-1-Beta vaccine complexes for 100 ns molecular dynamic simulation. ns = nanosecond. Coulombic SR = electrostatic short-range energy (in KJ/mol) within cutoff (approximately 0.9–1.2 nm). Epotential = Ebonded + Enonbonded. Bond energy = energy contribution from bond stretching between two covalently bonded atoms. (**a**) potential energy of vaccine-TLR2 complex over 100 ns, (**b**) potential energy of vaccine-TLR4 complex over 100 ns, (**c**) Culumbic short-range interaction of vaccine-TLR2 complex over 100 ns, (**d**) Culumbic short-range interaction of vaccine-TLR4 complex over 100 ns, (**e**) kinetic energy of vaccine-TLR2 complex over 100 ns, and, (**f**) kinetic energy of vaccine-TLR2 complex over 100 ns.

MMPBSA binding free energy (ΔG bind) calculated for the TLR2–vaccine and TLR4–vaccine complexes was −449.68 kJ/mol or −107.54 kcal/mol (TLR2–vaccine complex: −20,113.98 kcal/mol, vaccine: −6014.65 kcal/mol, and receptor: −13,991.79 kcal/mol) and −694.167 kJ/mol or −165.91 kcal/mol (TLR4–vaccine complex: −19,611.11 kcal/mol, vaccine: −5955.49 kcal/mol, and receptor: −13,489.70 kcal/mol). The MMPBSA findings suggest the thermodynamically favourable and stable interaction of the vaccine formulation with both the TLRs. Moreover, the molecular dynamics simulation energy profile exhibited a stable total potential energy for the TLR2/TLR4–vaccine (−2.15 × 10^6^ kJ/mol to −1.465 × 10^6^ kJ/mol) formulations with minor fluctuations (approximately ± 0.005 × 10^6^ kJ/mol), which suggests that the complex was stable without any potential drift over the simulation period (Figure 9a,b). Additionally, electrostatic/Culombic energy (electrostatic interaction between close atoms) was also computed for the TLR2/TLR4–vaccine complexes (~−2.695 × 10^6^ kJ/mol/~−1.905 × 10^6^ kJ/mol), conferring robust and stable electrostatic interaction over the whole simulation period (Figure 9c,d). Higher average kinetic energy was observed for the TLR-2/TLR4–vaccine complexes (2.74 × 10^5^ kJ/mol/3.9 × 10^5^ kJ/mol at 300K, inferring effective thermal stability and equilibrium throughout 100 ns (Figure 9e,f).

### 3.12. MPXV-1-Beta’s Immune Potency (Immune Simulation)

**Figure 10:**
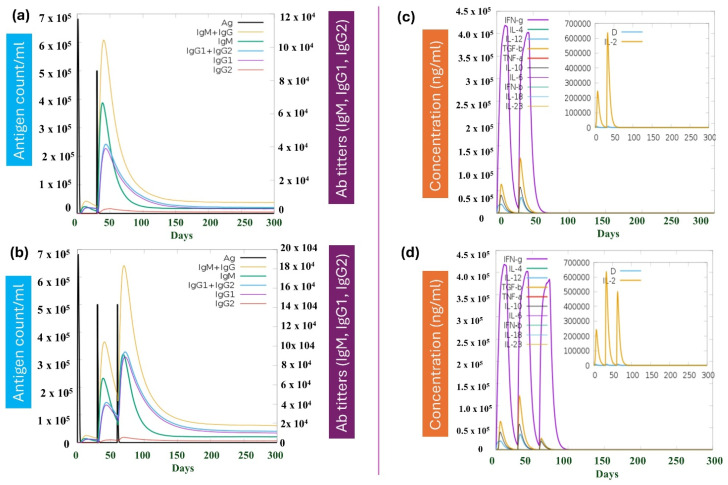
Immunological simulation results. (**a**) depicts the immune response (IR) over time (in days) after second injections after 30 days of MPXV-1-Beta; the black line denotes the antigen count/mL, which peaks rapidly around Day 1 and then declines swiftly as the IR eliminates the administered antigen after first dose. (**b**) The graph represents the cytokine and interleukin production dynamics over time (in days) after the second injection at 30 days. (**c**) The graph demonstrates the effect of a last (second booster) dose on Day 60. (**d**) exhibits the cytokine dynamics after the last (second booster dose) administered on Day 60.

Following the first antigen injection of the MPXV-1-Beta formulation, the antigen count peaked on Day 1 at approximately 7 × 10^5^ count/mL, followed by a swift dip in the antigen count, which signifies the activation of the immune system after the first dose. The initial IgM peak (7 × 10^4^) underscores the primary IR, followed by a rise in IgG levels (IgG1 and IgG2 (4 × 10^4^)), which remained elevated for longer, suggesting the induction of a sustained secondary IR after the second dose of the vaccine. The high IgG antibodies and efficient antigen clearance after injection explain the effective immunological memory formation (Figure 10a). Interferon-gamma (IFN-γ) exhibited a sustained response (4 × 10^5^ ng/mL). Interleukin-2 (IL-2) peaked (6.5 × 10^5^ ng/mL) and was significant in supporting T-cell proliferation and differentiation (Figure 10c). The cytokine-dynamic result showed a well-coordinated IR, where early activators (like IL-2) initiate IR, and later IFN-γ sustains immunological response (Figure 10c). Furthermore, the last booster dose on day 60 was finally administered to achieve a full-scale sustained primary and secondary IR against MPXV-1-Beta, which is depicted in Figure 10b. After the final (second) booster injection (Day 60), the antigen count peak (5 × 10^5^ count/mL) was observed; however, the count was lesser compared to the peak developed after the first antigen injection, signifying that the immune system was well-primed and speedily identified and eliminated the antigen. The total immunoglobulin response (IgM + IgG) attained after the final booster infection was approximately 18 × 10^4^ units. Additionally, IgM appeared first, followed by prolonged IgG elevation, suggesting the generation of immune memory (Figure 10b). Moreover, the IgG subclasses (IgG1, IgG2) exhibited sustained levels, highlighting adaptive immunity development and class-switching. Cytokine dynamic result suggested the interplay of pro-inflammatory and regulatory cytokines with IFN-γ (3.8 × 10^5^) and IL-2 (5.2 × 10^5^), which facilitates immune regulation (Figure 10d). The simulation exhibited the booster dose’s efficacy in prolonging IRs to provide long-term protection. The IRs elicited by MPXV-1-Beta vaccine formulation on the immunological cell population after each injection are depicted in Figures S4 and S5 of Supplementary File S2.

### 3.13. Optimisation of MPXV-1-Beta Codon and Gene Cloning

**Figure 11:**
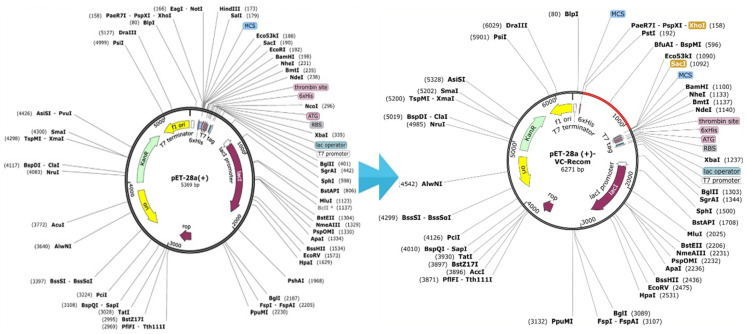
Cloning of multiepitope (MPXV-1-Beta). Insertion of multiepitope (MPXV-1-Beta) in 5369 bp vector (pET-28 (+)). Red-colour line represents multiepitope.

Before evaluating protein expression, optimising the codons for efficient protein synthesis is essential. The effectiveness of MPXV-1-Beta formulation expression is fundamental to successful vaccine development. The present research was conducted to evaluate MPXV-1-Beta protein expression in a bacterial strain (*E. coli* K12) (Figure S6; Supplementary File S3). The codon was successfully optimised (CAI;1.0, GC-content; 50.21367%) (Figure S6; Supplementary File S3). Restriction sites (EcoRI and XhoI, *n* = 02) were intentionally incorporated into the MPXV-1-Beta formulation’s N-terminal and C-terminal regions (Figure 9). Lastly, the proposed MPXV-1-Beta formulation was cloned, and a recombinant plasmid of 6271 base pairs was attained (Figure 11).

### 3.14. MPXV-1-Beta’s Population Coverage

**Figure 12:**
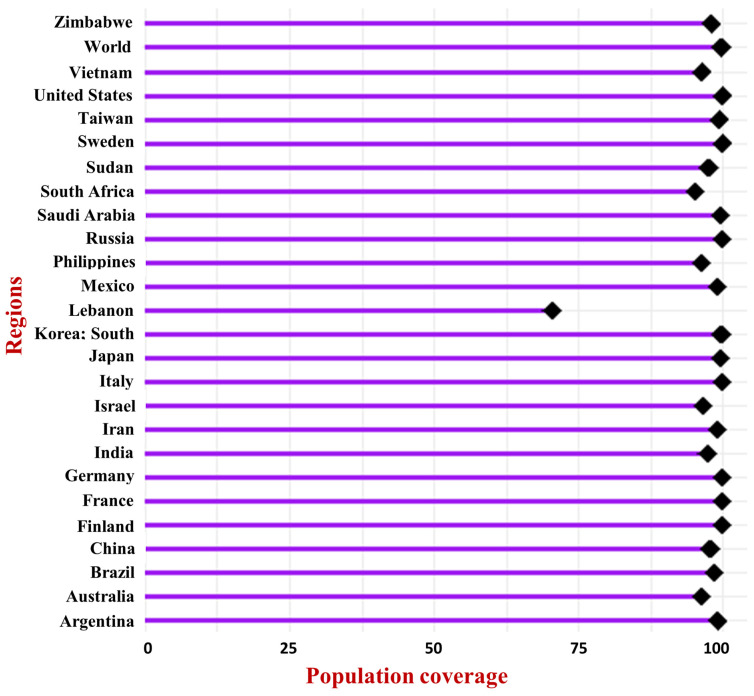
MPXV-1-Beta’s population coverage.

The population coverage analysis for the combined CTLEs and HTLEs demonstrated extensive global coverage. The selected epitopes achieved near-complete world coverage (99.74%), with an average worldwide coverage of 97.526 (SD = 12.44), underscoring their effectiveness in targeting a wide range of HLA alleles across various populations. This significant population coverage highlights the protective potential of MPXV-1-Beta formulation against hMPXV infection in diverse global populations, rendering MPXV-1-Beta a promising candidate for vaccination efforts (Figure 12).

## 4. Discussion

The recent monkeypox (hMPXV) outbreaks in non-endemic regions have caused an unexpected global health response, highlighting a significant deviation from previous patterns of the disease’s geographical distribution [18]. Outbreaks triggered by strains/sub-strains of hMPXV have posed considerable challenges in recent years. A definitive cure is lacking, and vaccines for hMPXV (currently in use) foster only limited protection [67]. Leveraging the combined potential of bioinformatics tools, a plethora of proteomic data, and reverse vaccinology vaccination, efforts can be accelerated to tackle current and future outbreaks [68]. In silico vaccine design exploits computational predictive modelling and proteomic data and provides a potential (free from ethical consideration, precision, cost reduction, efficiency, and safety enhancement) alternative to traditional methods of vaccine design, especially against rapidly mutating viruses [69]. These multiepitope vaccines recognise multiple HLA-epitopes by TCRs and elicit both humoral and cell-mediated IRs due to the incorporated epitopes (CTLE, HTLE, and LBE). Such vaccines can target a broader range of microbial antigenic components and allow the addition of suitable adjuvants to enhance IRs, giving them a clear edge over single-epitope vaccines [69].

Furthermore, with rare incorporation of undesirable microbial components, such vaccines exhibit minimum pathological IRs and immune-modulating responses. However, safety validation is required in the later developmental stage [70]. Given the absence of definitive treatment for hMPXV, the primary approach for managing outbreaks, primarily due to emerging strains/sub-strains, is preventive through first-line prophylactic measures. Considering these factors, the present study aimed to annotate crucial pathogenesis-related proteins (A35R and B6R) of the extracellular enveloped virus (EEV) form of hMPXV for designing a multiepitope vaccine and screening for the most suitable formulation against hMPXV.

For developing a complete vaccine formulation, the most suitable epitopes (LBEs, CTLEs, and HTLEs), which are critical components of a vaccine, were screened by targeting non-paralogous viral target proteins (A35R and B6R) [71]. Moreover, the strong evidence about the degree of conservancy of the selected epitopes across the strains/clades of the virus is paramount to providing broad-range protection; therefore, conservancy analyses play a pivotal role in in silico vaccine design [72]. Most of the epitopes included in this formulation were found to be 94% to 100% conserved across the strains/clades of hMPXV, which indicated the potential utility of the prioritised epitopes in designing vaccines that aim for broad cross-strain protection against hMPXV [73]. An autoimmune reaction with human protein is another major challenge for vaccine development; therefore, all the epitopes were processed on BLASTp against the human proteome, and the results did not exhibit any significant homology, underscoring the absence of an autoimmune reaction concern and strengthening the safety profile of the formulation [74]. BLASTp of hMPXV epitopes against Vaccinia virus protein is a necessary step to assess the off-target IRs and cross-reactivity because the Vaccinia virus is a closely related Orthopoxvirus, which could share some epitope sequences, leading to unintended immune activation or interference [23]. In the current research, lower identity (~59–67% identity) with partial query coverage (44–80%) to the Vaccinia virus proteome was observed, which reduces the likelihood of cross-reactive IR that could lead to off-target effects or reduce vaccine specificity. The BLASTp analysis approach corroborates with previous immunoinformatic analyses to screen candidate epitopes against closely related viruses [75].

In addition to epitopes, a CD4+ T-cell-activator, a non-natural pan-DR epitope with sequence AKVAAWTLKAAAC (binds to a broad range of HLA-DR molecules), was integrated into the MPXV-1-Beta sequence to MPXV-1-Beta to potentiate the formulation [76]. Moreover, MITD (providing a strong and reliable T-cell activation signal) is necessary for directing CTLEs to the MHC 1 compartment, and enhancing the antigen presentation was considered to be added [76]. Therefore, the MPXV-1-Beta formulation included the MITD sequence for boosting antigen presentation. Critical parameters (allergenicity and toxicity to the host) are pivotal in developing a safe vaccine formulation; thus, the epitopes/antigenic determinants included in the vaccine formulation were evaluated to exhibit nontoxicity and nonallergenicity to the host. Afterwards, the vaccine formulations were reassessed to achieve a safe and effective formulation [77]. Validating the antigenicity of each epitope and vaccine formulation at the initial developmental phase is highly significant; therefore, each epitope used for the final formulation and each vaccine formulation was studied to assess the antigenicity profile [77].

Adding adjuvants to vaccine constructs boosts IRs [70]. So, three different adjuvants incorporated to generate six formulations were further assessed for their suitability. All six formulations were inquisitively evaluated for their physicochemical properties, antigenicity score, nontoxicity attributes, nonallergenicity, and expression solubility to screen out the most promising formulation (MPXV-1-Beta). Studying the 3D structure of a protein is vital for evaluating its functionality (interactions with host immune receptor proteins required for activation of immunological pathways). Structural validation of the vaccine formulation ensures its functionality and stability over time. Thus, the secondary and 3D structure of MPXV-1-Beta formulation was studied and improved (refined). The ProSA (Z-score = −6.31) and Rama-favoured plus allowed region (98.8%) of the 3D model of MPXV-1-Beta signified that the formulation was structurally appropriate and thermodynamically stable for docking analysis. Additionally, MPXV-1-Beta was soluble, and the formulation’s solubility is paramount for the vaccine’s functionality [78].

A multiepitope MPXV-1-Beta formulation binding to TLRs is pivotal for activating the innate immune system, triggering the recruitment and maturation of immune cells, which enhances the subsequent adaptive IRs [79]. Therefore, docking was accomplished to determine the interaction of the proposed MPXV-1-Beta formulation with TLRs on immune cells, which identify the pathogen-associated molecular patterns (PAMPs), triggering innate immune signalling leading to the elicitation of adaptive IRs [79]. NMA is vital for examining biomolecular complexes’ stability, flexibility, and molecular motion during dynamic interaction, ensuring their functional integrity [80]. NMA’s finding indicated that TLR4-MPXV-1-Beta was a stable conformation with an extremely low eigenvalue (0.48392 × 10^−4^), which implies that TLR4-MPXV-1-Beta is allowed for considerable collective motions, significant for functional flexibility. Docking and NMA findings suggest the strong and stable interaction of TLR4 with MPXV-1-Beta [80]. A receptor (TLR2) fosters a protective role by recognising the viral elements and initiating a pro-inflammatory response [80,81]. The eigenvalues (0.149178 × 10^−4^) of TLR2-MPXV-1-Beta suggest that the MPXV-1-Beta formulation interacted strongly with the receptor, leading to a stable/rigid molecular interaction [82]. NMA of MPXV-1-Beta with MHC (class I and class II) molecules also showed molecular interaction with negative relative eigenvalues (0.63157 × 10^−4^) for class I and (0.44575 × 10^−4^) for class II. Generating a highly stable MPXV-1-Beta-MHC complex during NMA signifies that the formulation could trigger adaptive IRs [83]. The MDS provides key insights into the stability of the MPXV-1-Beta vaccine formulation when bound to LR2 and TLR4 [84]. Both complexes exhibited the RMSD values stabilising within the first 10 ns of the 100 ns simulation. These stable RMSD profiles of TLR2/TLR4–vaccine complexes suggest that the structural integrity of both complexes was preserved, with no signs of unfolding or potential deviation, which is a pivotal factor for determining consistent biological activity over time [84]. RMSF analyses (to monitor the flexibility of the individual residue within the TLR–vaccine complex during simulation) revealed that the majority of residues remained relatively rigid, with small fluctuations suggesting stable conformations over time. Additionally, this research analysed the radius of gyration (to assess compactness) and hydrogen bonds (to monitor the interaction dynamics between TLRs and the vaccine construct), which revealed compactness and strong intermolecular interactions (consistent high count: 600–680) between TLR–vaccine complexes reflecting robust vaccine–receptor binding in biological systems supported by MMPBSA binding free energy (ΔGbind) of this research for TLR2/TL4-vaccine complexes, which corroborates findings reported in other studies [85,86].

Additionally, the decreasing trend in SASA observed during the simulation implies that both complexes became more tightly packed over simulation time, reducing solvent exposure and increasing compaction, which reflects the complexes’ structural stability required for antigenic presentation [87]. Energetically, the total potential energy remained steady for both complexes, suggesting that the systems were well-equilibrated and structurally sound. The Coulombic short-range interaction energy also remained consistent, reflecting the sustained electrostatic attraction between charged residues, which is necessary for molecular recognition and complex formation [88]. Moreover, both complexes exhibited stable kinetic energy at 300K during simulation, reflecting thermal stability. Overall, MDS revealed the thermodynamically favourable interaction between the TLR-MPXV-1-Beta construct, suggesting a high potential for the construct to undergo further experimental validation and preclinical evaluation.

The *E. coli* K12 strain is frequently utilised to produce recombinant proteins on a large scale, and achieving efficient expression requires codon adaptation [89]. The proposed MPXV-1-Beta formulation, in this research, achieved a CAI of 1.0 and GC content of 50.21367%, which is appropriate for high yield because optimal expression could be achieved with CAI > 0.890 and GC content in the range of 30–70% [90]. Immunological simulation of MPXV-1-Beta formulation is significant in identifying the most promising candidate before clinical trials, fostering a reduction in time and cost-effective vaccine development [91]. After completing the boosters, the proposed formulation successfully elicited both arms of IRs (Figure 8). Assessing the efficacy of MPXV-1-Beta formulation across diverse demographics is one of the significant indicators of the designed vaccine [91]. This study evaluated the MPXV-1-Beta formulation’s efficacy in eliciting IRs in geographically distinct individuals. Combined world population coverage was computed to be remarkable (99.74%).

## 5. Limitation

This detailed evaluation examined the multiepitope formulation MPXV-1-Beta as a potential candidate, though certain limitations persist. Despite being rapid and cost-effective, the reverse vaccinology approach heavily relies on a predictive algorithm, which may not be translated into in vivo IRs completely, which highlights the uncertainty in the proposed protection [92]. Lacking experimental validation (in vitro and in vivo) limits the confirmation of antigen processing, epitope antigenicity, and safety of the proposed vaccine in humans [93]. Moreover, population-specific variability (HLA-variability) and variation in the viral genome can influence vaccine efficacy, necessitating further laboratory and clinical investigation to corroborate in silico results [94]. The challenges include insufficient benchmarking, restricted predictive approaches, and inadequate datasets. Although recent successes have been with multiepitope vaccine formulations, especially against COVID-19, the MPXV-1-Beta formulation necessitates in vitro and in vivo bioassay studies to validate its safety and efficacy.

## 6. Conclusions

A multiepitope vaccine against hMPXV is crucial in the current scenario to provide broad, adequate protection and address the growing necessity for rapid, adaptable solutions to emerging hMPXV strains’ threats. This research harnessed the reverse vaccinology and immunoinformatic approaches to propose a novel multiepitope vaccine formulation to combat hMPXV outbreaks. At first, six formulations were generated by combining different epitopes and adjuvants. One (MPXV-1-Beta) was screened as the most appropriate candidate for further analysis based on all six formulations’ physicochemical properties and antigenicity. Docking, followed by NMA of MPXV-1-Beta with TLRs and MHC molecules, signified stable MPXV-1-Beta–immune receptor (TLRs and MHCs) molecular interaction to generate sustained and robust IRs. Moreover, the 100 ns MDS for TLR–vaccine complexes, followed by MM/PBSA binding energy assessment, further validated the TLR–vaccine complexes’ stability. MPXV-1-Beta, the optimal formulation, demonstrated strong immunogenicity, nontoxicity, nonallergenicity, TLR binding affinity, and stable gene expression in *E. coli*. Moreover, the average population coverage of MPXV-1-Beta was remarkably high (97.526, SD = 12.44). Though the MPXV-1-Beta formulation presented a promising first-line prophylactic effect for curbing future hMPXV outbreaks, validation of the current findings through experimental and clinical studies is sought to authenticate the safety and efficacy of the proposed formulation.

## Figures and Tables

**Table 1:** Baseline characteristics (physicochemical) of the target MPXV proteins’ sequences for vaccine formulation.

Parameters	A35R/EEV	B6R/EEV
Allergenicity	Non-allergen	Non-allergen
Antigenicity/score	Ag/0.4976	Ag/0.5786
Residue toxicity	Nontoxic	Nontoxic
AA Length	181	317
Mw/TpI	20,050.55/5.59	35,145.87/4.67
AI	73.81	77.98
GRAVY	−0.305	−0.182
Ii	42.34	41.79
EHL-MR	30 h	30 h
EHL-Y	>20 h	>20 h
EHL-E	>10 h	>10 h
EC (M^−1^cm^−1^)	24,410	42,860
E. solubility	0.719	0.557

EEV = extracellular enveloped virus; AA = amino acid; Ag = antigenic; Mw = molecular weight; TpI = theoretical pI; pI = isoelectric point; AI = aliphatic index, grand average of hydropathicity; Ii = instability index; EHL-MR = estimated half-life in mammalian reticulocytes or in vitro; EHL-Y = estimated half-life in yeast or in vivo; EHL-E = estimated half-life in *E. coli* or in vivo; E. solubility = expression solubility; and EC = extinction coefficient measured in water at 280.

**Table 2:** The toxicity, immunogenic potential, and allergenicity of the prioritised LBEs and CTLEs.

Proteins IDs/Proteins	LBL-Epitope Designation	LBL-Epitope	ABCP(s)	Ag(s)	Toxicity	Allergenicity
QJQ40286.1/A35R/EEV	LBE-1/A35R	VVSSTTQYDHKESCNG	0.9	0.8364	NT	NA
	LBE-2/A35R	TKTTSDYQDSDVSQEV	0.88	0.7943	NT	NA
	LBE-3/A35R	CIRISMVISLLSMITM	0.58	1.1317	NT	NA
AAL40625.1/B6R/EEV	LBE-4/B6R	PTCVRSNEEFDPVDDG	0.88	1.1564	NT	NA
	LBE-5/B6R	KLTSTETSFNDKQKVT	0.83	1.199	NT	NA
	LBE-6/B6R	TGSPSSTCIDGKWNPI	0.83	1.059	NT	NA
Proteins IDs/Proteins	CTL-epitope designation	CTL-epitope	IC_50_-value	Ag(s)	Toxicity	Allergenicity
QJQ40286.1/A35R/EEV	CTL-1/A35R	GLCIRISMV	23.45	2.0951	NT	NA
	CTL-2/A35R	AAASSTHRK	72.79	1.0499	NT	NA
	CTL-3/A35R	RISMVISLL	86.55	0.9423	NT	NA
AAL40625.1/B6R/EEV	CTL-4/B6R	CIDGKWNPI	85.98	1.734	NT	NA
	CTL-5/B6R	ETSFNDKQK	24.5	1.4922	NT	NA
	CTL-6/B6R	STETSFNDK	58.49	1.441	NT	NA

EEV = extracellular enveloped virus; LBL = linear B-cell; ABCP(s) = ABCPred score; Ag(s) = antigenicity score (Vaxijen score); NT = nontoxic; and NA = nonallergenic.

**Table 3:** The immunogenic potential of MHC-class-2 binders (IL-4 inducer overlapped) and IFN-gamma epitopes.

Proteins IDs/Proteins	HTL-Epitope Designation	HTL-Epitope	IC_50_-Value	Ag(s)	II/IL-4 Score	Tox/Aller
QJQ40286.1/A35R/EEV	HTL-1/A35R	KRKRVIGLCIRISMV	36.1	1.8051	0.29	NT/NA
	HTL-2/A35R	RKRVIGLCIRISMVI	56.3	1.5987	0.27	NT/NA
	HTL-3/A35R	GKNKRKRVIGLCIRI	14.3	1.3714	0.29	NT/NA
AAL40625.1/B6R/EEV	HTL-4/B6R	NAKLTSTETSFNDKQ	90.9	1.4473	1.16	NT/NA
	HTL-5/B6R	DSGYHSLDPNAVCET	30.7	0.9057	0.28	NT/NA
	HTL-6/B6R	DGKWNPILPTCVRSN	9.6	0.6373	1.33	NT/NA
Proteins IDs/Proteins	IFN-γ-epitope designation	IFN-gamma Epitope	IC_50_-value	Ag(s)	IFN-γ scores	Tox/Aller
QJQ40286.1/A35R/EEV	IFN-γ-1/A35R	SMVISLLSMITMSAF	80.6	0.573	1.267	NT/NA

EEV = extracellular enveloped virus, IFN-γ = interferon-gamma, HTL = helper T-cell, Ag(s) = antigenicity score (Vaxijen), IL-4 = interleukin-r, II = IL-4-inducing, Tox = toxicity, Aller = allergenicity, NT = non-toxic, and NA = non-allergenic.

**Table 4:** Epitope conservancy across hMPXV strains, including Clade I and Clade II.

Epitope Sequence	Epitope Length	Percent of Protein Sequence Matches at Identity ≤ 100%	Minimum Identity	Maximum Identity
VVSSTTQYDHKESCNG	16	100.00% (30/30)	93.75%	100.00%
TKTTSDYQDSDVSQEV	16	100.00% (30/30)	100.00%	100.00%
CIRISMVISLLSMITM	16	100.00% (30/30)	100.00%	100.00%
GLCIRISMV	9	100.00% (30/30)	100.00%	100.00%
AAASSTHRK	9	100.00% (30/30)	100.00%	100.00%
RISMVISLL	9	100.00% (30/30)	100.00%	100.00%
KRKRVIGLCIRISMV	15	100.00% (30/30)	100.00%	100.00%
RKRVIGLCIRISMVI	15	100.00% (30/30)	100.00%	100.00%
GKNKRKRVIGLCIRI	15	100.00% (30/30)	100.00%	100.00%
SMVISLLSMITMSAF	15	100.00% (30/30)	100.00%	100.00%
PTCVRSNEEFDPVDDG	16	89.74% (35/39)	25.00%	100.00%
KLTSTETSFNDKQKVT	16	89.74% (35/39)	18.75%	100.00%
TGSPSSTCIDGKWNPI	16	87.18% (34/39)	18.75%	100.00%
CIDGKWNPI	9	89.74% (35/39)	33.33%	100.00%
ETSFNDKQK	9	89.74% (35/39)	33.33%	100.00%
STETSFNDK	9	89.74% (35/39)	33.33%	100.00%
NAKLTSTETSFNDKQ	15	89.74% (35/39)	26.67%	100.00%
DSGYHSLDPNAVCET	15	89.74% (35/39)	20.00%	100.00%
DGKWNPILPTCVRSN	15	89.74% (35/39)	33.33%	100.00%

**Table 5:** Salient characteristics of six (*n* = 06) different vaccine formulations/constructs.

Parameters	MPXV-1- Hbha	MPXV-1- Beta	MPXV-1- Ribo	MPXV-2-Hbha	MPXV-2-Beta	MPXV-2-Ribo
Allergenicity	Non-allergen	Non-allergen	Non-allergen	Non-allergen	Non-allergen	Non-allergen
Antigenicity/score	Ag/0.712	Ag/0.7953	Ag/0.6938	Ag/0.7596	Ag/0.6859	Ag/0.6659
Residue toxicity/score	Nontoxic/0.16	Nontoxic/0.27	Nontoxic/0.27	Nontoxic/0.2	Nontoxic/0.17	Nontoxic/0.2
AA Length	426	312	397	313	427	398
Mw/TpI	44,981.24/8.7	32,513.79/9.76	40,793.07/8.77	32,427.54/9.48	44,894.99/6.13	40,706.83/6.33
AI	81.27	74.23	84.71	55.97	67.87	70.33
Gravy	−0.248	−0.187	−0.009	−0.539	−0.506	−0.286
Ii	34	30.62	25	29.36	33.17	24.62
EHL-MR	1 h	1 h	1 h	1 h	1 h	1 h
EHL-Y	30 min	30 min	30 min	30 min	30 min	30 min
EHL-E	>10 h	>10 h	>10 h	>10 h	>10 h	>10 h
EC (M^−1^cm^−1^)	31,775	30,660	27,305	37,525	38,640	34,170
E. solubility	0.698	0.920	0.660	1.138	0.855	0.833
E. solubility/suloscore	ISE/0.185	SE/0.830	ISE/0.42	SE/0.883	ISE/0.398	SE/0.612

AA = amino acid; Ag = antigenic; Mw = molecular weight; TpI = theoretical pI; AI = aliphatic index; Gravy = grand average of hydropathicity; Ii = instability index; EHL-MR = estimated half-life in mammalian reticulocytes or in vitro; EHL-Y = estimated half-life in Yest or in vivo; EHL-E = estimated half-life in *E. coli* or in vivo; E. solubility = expression solubility; SE = soluble expression; ISE = insoluble expression; suloscore cutoff > 0.5; and EC = extinction coefficient measured in water at 280.

**Table 6:** Summary of the interacting amino acid residues, polar contacts, and interface area in TLRs-MPXV-1-Beta and MHCs-MPXV-1-Beta complexes.

Complex Description	No. of Residues in the Interface	Count of Salt Bridges	Count of Hydrogen Bonds	Count of Non-Bonded Contacts	Interface Area (Å^2^)
TLR-4	38	04	22	198	1627
MPXV-1-Beta	32				1792
TLR-2	22	04	19	135	1050
MPXV-1-Beta	19				1125
MHC-I-MPXV-1-Beta-Complex	23–23 (A–C)	07 (A–C)	10 (A–C)	143 (A–C)	1198–1256 (A–C)
	05–06 (B–C)	03 (B–C)	03 (A–C)	31 (A–C)	353–329 (B–C)
MHC-II-MPXV-1-Beta-Complex	19–23 (A–C)	06 (A–C)	13 (A–C)	154 (A–C)	1062–1017 (A–C)
	15–17 (B–C)	0 (B–C)	05 (B–C)	97 (B–C)	802–823 (B–C)

A = chain A of MHC class-I and chain B of MHC class-II. C = chain of proposed MPXV-1-Beta formulation.

## Data Availability

All original materials (materials) and methods are available in the article and Supplementary Documents; however, the corresponding authors could be contacted for further inquiries.

## Supplementary Materials

The following supporting information can be downloaded at: https://www.mdpi.com/article/10.3390/biology14070830/s1, Supplementary File S1 (Figure S1. CD-Hit clusters and sequences of the target protein with their accession numbers (A35R: Cluster 1 and B6R: cluster 2), Figure S2. Secondary structure elements of the MPXV-1-Beta formulation and Figure S3. mRNA secondary structure of the MPXV-1-Beta and minimum free energies), Supplementary File S2 (Figure S4. Results of Immune simulation after the second booster dose and Figure S5. Effect of proposed MPXV-1-Beta formulation on populations of immune cells after final booster dose), and Supplementary File S3 (Figure S6. Codon adaptation results).
