# Supplementary material for: Annotation of the Extracellular Enveloped Form of Monkeypox Virus for the Design, Screening, Validation, and Simulation of a Chimeric Vaccine Construct
Source: Biology (Basel). 2025 Jul 8;14(7):830. doi: 10.3390/biology14070830 (PMC12292176; doi:10.3390/biology14070830)
Supplement: Supplementary file 1 [file biology-14-00830-s001.zip › Supplementary File S3.pdf]

Bioinformatic tools from our team:

PRODORIC Release 2

JCat was published in NAR (Nucleic Acids Research).

# CodonUsage adapted to Escherichia coli (strain K12)

Improved DNA:

```
GAAGCTGCTGCTAAAGGTATCATCAACACCCTGCAGAAATACTACTGCCG      50
TGTTCGTGGTGGTCGTTGCGCTGTTCTGTCTTGCCTGCCGAAAGAAGAAC      100
AGATCGGTAAATGCTCTACCCGTGGTCGTAAATGCTGCCGTCGTAAAAAA      150
GAAGCTGCTGCTAAAGCTAAATTCGTTGCTGCTTGGACCCTGAAAGCTGC      200
TGCTGGTGGTGGTTCTGTTGTTTCTTCTACCACCCAGTACGACCACAAAG      250
AATCTTGCAACGGTGGTCCGGGTCCGGGTAAAAAAAAACGTAAACGTGTT      300
ATCGGTCTGTGCATCCGTATCTCTATGGTTGCTGCTTACGGTCTGTGCAT      350
CCGTATCTCTATGGTTGGTGGTGGTTCTCACGAATACGGTGCTGAAGCTC      400
TGGAACGTGCTGGTGGTGGTGGTTCTCCGACCTGCGTTCGTTCTAACGAA      450
GAATTCGACCCGGTTGACGACGGTGGTCCGGGTCCGGGTAAAAAAAAACGC      500
TAAACTGACCTCTACCGAAACCTCTTTCAACGACAAAGCTGCTTACTGCA      550
TCGACGGTAAATGGAACCCGATCGGTGGTGGTTCTCACGAATACGGTGCT      600
GAAGCTCTGGAACGTGCTGGTGGTGGTGGTTCTTGCATCCGTATCTCTAT      650
GGTTATCTCTCTGCTGTCTATGATCACCATGGGTCCGGGTCCGGGTAAAA      700
AAGGTAAAAACAAACGTAAACGTGTTATCGGTCTGTGCATCCGTATCGCT      750
GCTTACCGTATCTCTATGGTTATCTCTCTGCTGGGTCCGGGTCCGGGTAA      800
AAAATCTATGGTTATCTCTCTGCTGTCTATGATCACCATGTCTGCTTTCC      850
ACGAATACGGTGCTGAAGCTCTGGAACGTGCTGGTGCTAAATTCGTTGCT      900
GCTTGGACCCTGAAAGCTGCTGCTGGTGGTGGTTCT
```

CAI-Value of the improved sequence:

1.0

GC-Content of the improved sequence:

50.21367521367522

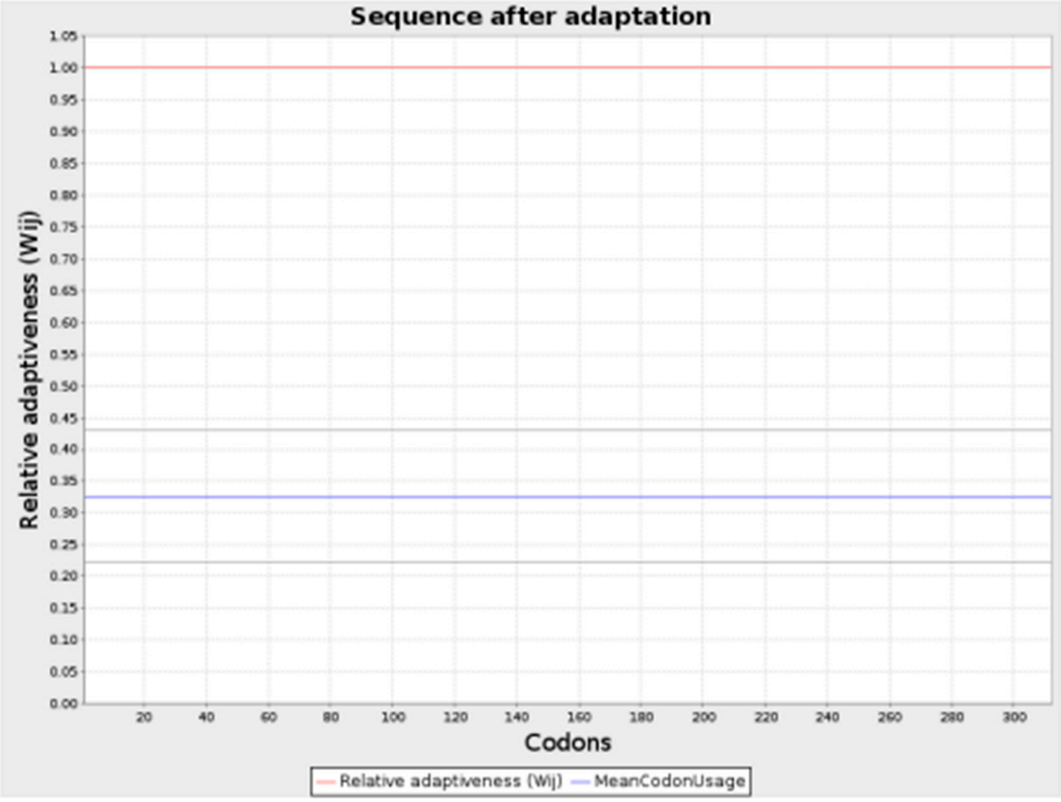


Detailed Table View!

**Improved DNA:**

```
GAAGCTGCTGCTAAAGGTATCATCAACACCCTGCAGAAATACTACTGCCG     50
TGTTCGTGGTGGTCGTTGCGCTGTTCTGTCTTGCCTGCCGAAAGAAGAAC     100
AGATCGGTAAATGCTCTACCCGTGGTCGTAAATGCTGCCGTCGTAAAAAA     150
GAAGCTGCTGCTAAAGCTAAATTCGTTGCTGCTTGGACCCTGAAAGCTGC     200
TGCTGGTGGTGGTTCTGTTGTTTCTTCTACCACCCAGTACGACCACAAAG     250
AATCTTGCAACGGTGGTCCGGGTCCGGGTAAAAAAAAACGTAAACGTGTT     300
ATCGGTCTGTGCATCCGTATCTCTATGGTTGCTGCTTACGGTCTGTGCAT     350
CCGTATCTCTATGGTTGGTGGTGGTTCTCACGAATACGGTGCTGAAGCTC     400
TGGAACGTGCTGGTGGTGGTGGTTCTCCGACCTGCGTTCGTTCTAACGAA     450
GAATTCGACCCGGTTGACGACGGTGGTCCGGGTCCGGGTAAAAAAAAACGC     500
TAAACTGACCTCTACCGAAACCTCTTTCAACGACAAAGCTGCTTACTGCA     550
TCGACGGTAAATGGAACCCGATCGGTGGTGGTTCTCACGAATACGGTGCT     600
GAAGCTCTGGAACGTGCTGGTGGTGGTGGTTCTTGCATCCGTATCTCTAT     650
GGTTATCTCTCTGCTGTCTATGATCACCATGGGTCCGGGTCCGGGTAAAA     700
AAGGTAAAAACAAACGTAAACGTGTTATCGGTCTGTGCATCCGTATCGCT     750
GCTTACCGTATCTCTATGGTTATCTCTCTGCTGGGTCCGGGTCCGGGTAA     800
AAAATCTATGGTTATCTCTCTGCTGTCTATGATCACCATGTCTGCTTTCC     850
ACGAATACGGTGCTGAAGCTCTGGAACGTGCTGGTGCTAAATTCGTTGCT     900
GCTTGGACCCTGAAAGCTGCTGCTGGTGGTGGTTCT
```

## Translation

```
EAAAKGIINTLQKYYCRVRGGRCAVLSCLPKEEQIGKCSTRGRKCCRRKK     50
EAAAKAKFVAAWTLKAAAGGGSVVSSTTQYDHKESCNGGPGPGKKKRKRV     100
IGLCIRISMVAAYGLCIRISMVGGGSHEYGAEALERAGGGGSPTCVRSNE     150
EFDPVDDGGPGPGKKNAKLTSTETSFNDKAAYCIDGKWNPIGGGSHEYGA     200
EALERAGGGGSCIRISMVISLLSMITMGPGPGKKGKNKRKRVIGLCIRIA     250
AYRISMVISLLGPGPGKKSMVISLLSMITMSAFHEYGAEALERAGAKFVA     300
AWTLKAAAGGGS
```

**Figure S6. codon adaptation results**
