# Supplementary material for: Annotation of the Extracellular Enveloped Form of Monkeypox Virus for the Design, Screening, Validation, and Simulation of a Chimeric Vaccine Construct
Source: Biology (Basel). 2025 Jul 8;14(7):830. doi: 10.3390/biology14070830 (PMC12292176; doi:10.3390/biology14070830)
Supplement: Supplementary file 1 [file biology-14-00830-s001.zip › Supplementary File S1.pdf]

```
>Cluster 0
0       212aa, >fig|10244.7281.CDS.... *
1       212aa, >fig|10244.7292.CDS.... at 100.00%
2       212aa, >fig|10244.7325.CDS.... at 100.00%
3       212aa, >fig|10244.7326.CDS.... at 100.00%
4       212aa, >fig|10244.7328.CDS.... at 100.00%
5       212aa, >fig|10244.7342.CDS.... at 100.00%
6       212aa, >fig|10244.786.CDS.3... at 100.00%
>Cluster 1
0       181aa, >QJQ40286.1... *
1       181aa, >AGR38704.1... at 98.90%
2       181aa, >AGR38513.1... at 98.90%
3       181aa, >AGR38322.1... at 98.90%
4       181aa, >AGR38131.1... at 98.90%
5       181aa, >AGR37941.1... at 98.90%
6       181aa, >AGR37751.1... at 98.90%
7       181aa, >AGR37561.1... at 98.90%
8       181aa, >AGR37370.1... at 98.90%
9       181aa, >AGR37179.1... at 98.90%
10      181aa, >AGR36988.1... at 98.90%
11      181aa, >AGR36797.1... at 98.90%
12      181aa, >AGR36606.1... at 98.90%
13      181aa, >AGR36415.1... at 98.90%
14      181aa, >AGR36224.1... at 98.90%
15      181aa, >AGR36033.1... at 98.90%
16      181aa, >AGR35842.1... at 98.90%
17      181aa, >AGR35651.1... at 98.90%
18      181aa, >AGR35460.1... at 98.90%
19      181aa, >AGR35269.1... at 98.90%
20      181aa, >AGR35078.1... at 98.90%
21      181aa, >AGR34887.1... at 98.90%
22      181aa, >AGR34696.1... at 98.90%
23      181aa, >AGR34506.1... at 98.90%
24      181aa, >AAL40603.1... at 98.34%
25      181aa, >AAN78222.1... at 98.90%
26      181aa, >USS79496.1... at 100.00%
27      181aa, >AKG51495.1... at 98.90%
28      181aa, >AKG51308.1... at 98.90%
```

CD-Hit cluster QJQ40286.1/A35R/EEV

```
>Cluster 0
0       1879aa, >fig|10244.1778.CDS.... at 99.04%
1       1880aa, >fig|10244.1829.CDS.... *
2       1880aa, >fig|10244.3341.CDS.... at 100.00%
3       1880aa, >fig|10244.6951.peg.... at 100.00%
4       1880aa, >fig|10244.6952.CDS.... at 100.00%
5       1880aa, >fig|10244.714.CDS.1... at 100.00%
6       1880aa, >fig|10244.8238.CDS.... at 100.00%
7       64aa, >fig|10244.8278.peg.... at 98.44%
8       59aa, >fig|10244.8278.peg.... at 100.00%
9       46aa, >fig|10244.8278.peg.... at 100.00%
10      58aa, >fig|10244.8282.peg.... at 100.00%
11      70aa, >fig|10244.8282.peg.... at 100.00%
12      49aa, >fig|10244.8282.peg.... at 100.00%
13      49aa, >fig|10244.8282.peg.... at 100.00%
14      50aa, >fig|10244.8282.peg.... at 100.00%
15      70aa, >fig|10244.8282.peg.... at 100.00%
16      49aa, >fig|10244.8282.peg.... at 100.00%
17      99aa, >fig|10244.8282.peg.... at 100.00%
18      49aa, >fig|10244.8282.peg.... at 100.00%
19      97aa, >fig|10244.8282.peg.... at 100.00%
20      1879aa, >fig|10244.9305.CDS.... at 98.99%
21      1247aa, >fig|10244.9543.CDS.... at 99.36%
>Cluster 1
0       322aa, >fig|10244.714.CDS.1... *
>Cluster 2
0       317aa, >AAL40625.1... *
1       317aa, >AAN78223.1... at 100.00%
2       317aa, >QJQ40306.1... at 100.00%
3       317aa, >AKG51517.1... at 100.00%
4       317aa, >AKG51329.1... at 100.00%
5       317aa, >AKG51144.1... at 100.00%
6       317aa, >AGR38726.1... at 100.00%
7       317aa, >AGR38535.1... at 100.00%
8       317aa, >AGR38344.1... at 100.00%
9       317aa, >AGR38153.1... at 100.00%
10      317aa, >AGR37963.1... at 100.00%
11      317aa, >AGR37773.1... at 100.00%
```

CD-Hit cluster AAL40625.1/B6R/EEV

```
>QJQ40286.1 A35R [Monkeypox virus]
MMTPENDEEQTSVFSATVYGDKIQGKNKRKRVIGLCIRISMVISLLSMITMSAFLIVRLNQCMSANKAAI
TDSAVAVAAASSTHRKVVSSTTQYDHKESCNGLYYQGSCYILHSDYKSFEDAKANCAAESSTLPNKSDVL
TTWLIDYVEDTWGSDGNPITKTTSDYQDSDVSQEVRKYFCT

>AAL40625.1 B6R [Monkeypox virus Zaire-96-I-16]
MKTISVVTLLCVLPAVVYSTCTVPTMNNAKLTSTETSFNDKQKVTFTCDSGYHSLDPNAVCETDKWKYEN
PCKKMCTVSDYVSELYDKPLYEVNSTMTLSCNGETKYFRCEEKNGNTSWNDTVTCPNAECQPLQLEHGSC
QPVKEKYSFGEYMTINCDVGYEVIGVSYISCTANSWNVIPSCQQKCDIPSLSNGLISGSTFSIGGVIHLS
CKSGFTLTGSPSSTCIDGKWNPILPTCVRSNEEFDPVDDGPDDETDLSKLSKDVVQYEQEIESLEATYHI
IIMALTIMGVIFLISIIVLVCSCDKNNDQYKFHKLLP
```

Pruned target protein sequence for down-stream analysis

Figure S1. CD-Hit clusters and sequences of the target protein with their accession numbers (A35R: Cluster 1 and B6R: cluster 2)

SOPMA result for : UNK_12639810

Abstract Geourjon, C. & Deléage, G., SOPMA: Significant improvement in protein secondary structure prediction by consens

```
         10        20        30        40        50        60        70
          |         |         |         |         |         |         |
EAAAKGIINTLQKYYCRVRGGRCAVLSCLPKEEQIGKCSTRGRKCCRRKKEAAAKAKFVAAWTLKAAAGG
hhhhhhhhhhhhhhhhhccccche eeeeccccccccеeccccccccccchhhhhhhhhhhhhhhhhhhccc
GSVVSSTTQYDHKESCNGGPGPGKKKRKRVIGLCIRISMVAAYGLCIRISMVGGGSHEYGAEALERAGGG
cceeeeccccccccccccccccccccceeeeeeeeeehhhhccceeeeeeecccccchhhhhhhhhcccc
GSPTCVRSNEEFDPVDDGGPGPGKKNAKLTSTETSFNDKAAYCIDGKWNPIGGGSHEYGAEALERAGGGG
cccceeeccccccccccccccccccccceeecccccccccheeeecccccccccccchhhhhhhhhccccc
SCIRISMVISLLSMITMGPGPGKKGKNKRKRVIGLCIRIAAYRISMVISLLGPGPGKKSMVISLLSMITM
ceeeeeeehhhheeecccccccccccccheeeeeeeeeehhhhheeeeeeecccccccceeeeehhhhhhh
SAFHEYGAEALERAGAKFVAAWTLKAAAGGGS
hhhhhhhhhhhhhhhhhhhhhhhhhhhccccc

Sequence length :   312

SOPMA :
   Alpha helix      (Hh) :   105 is  33.65%
   3_10 helix        (Gg) :     0 is   0.00%
   Pi helix          (Ii) :     0 is   0.00%
   Beta bridge       (Bb) :     0 is   0.00%
   Extended strand  (Ee) :    69 is  22.12%
   Beta turn         (Tt) :     0 is   0.00%
   Bend region       (Ss) :     0 is   0.00%
   Random coil       (Cc) :   138 is  44.23%
   Ambiguous states (?)  :     0 is   0.00%
   Other states           :     0 is   0.00%
```

Figure S2. Secondary structure elements of the MPXV-1-Beta formulation

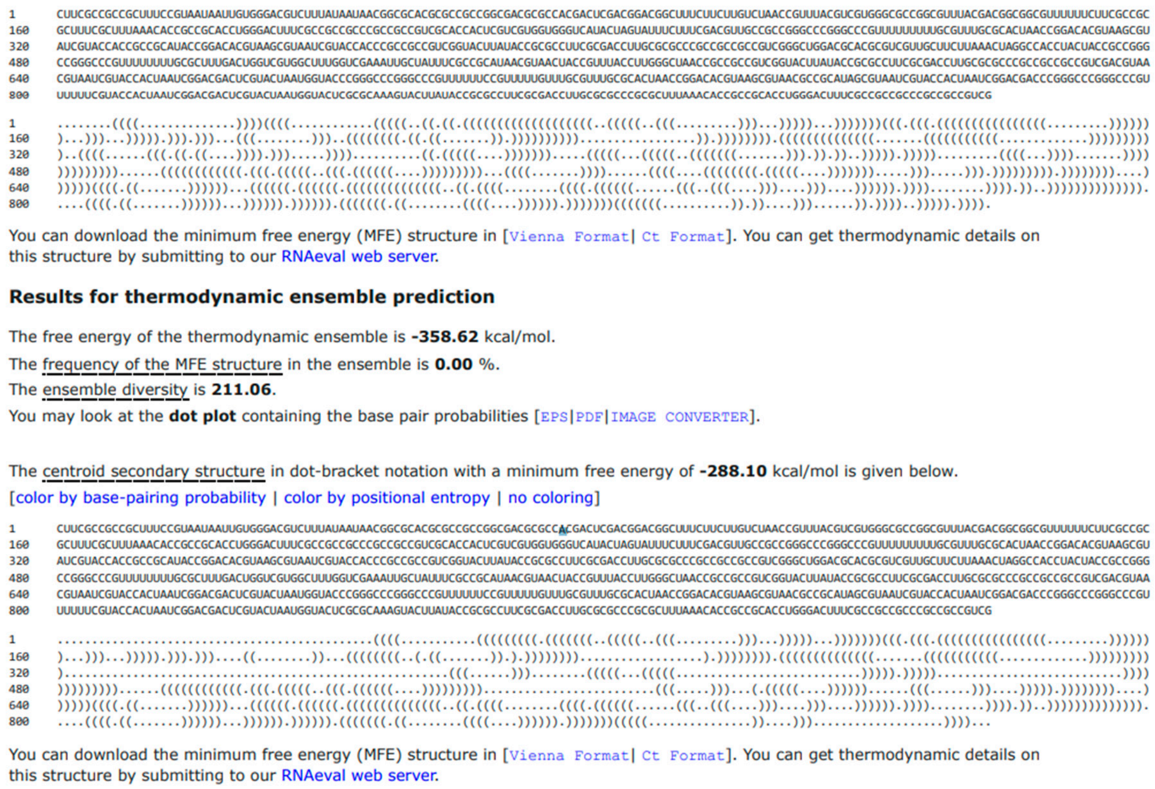

Figure S3. mRNA secondary structure of the MPXV-1-Beta and minimum free energies.
