# Supplementary material for: Annotation of the Extracellular Enveloped Form of Monkeypox Virus for the Design, Screening, Validation, and Simulation of a Chimeric Vaccine Construct
Source: Biology (Basel). 2025 Jul 8;14(7):830. doi: 10.3390/biology14070830 (PMC12292176; doi:10.3390/biology14070830)
Supplement: Supplementary file 1 [file biology-14-00830-s001.zip › Supplementary File S2.pdf]

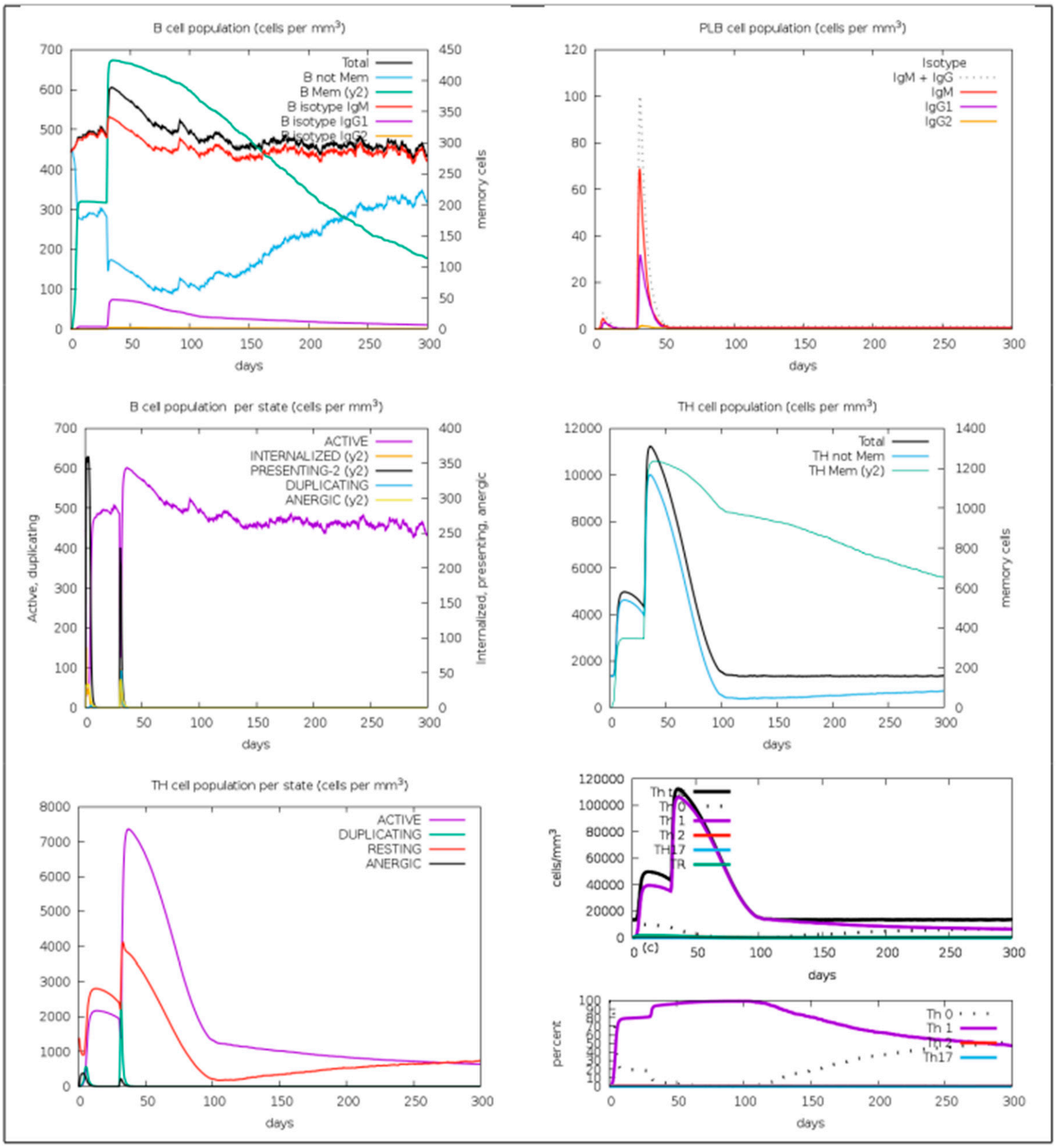

Effect of proposed MPXV-1-Beta on B-cell population

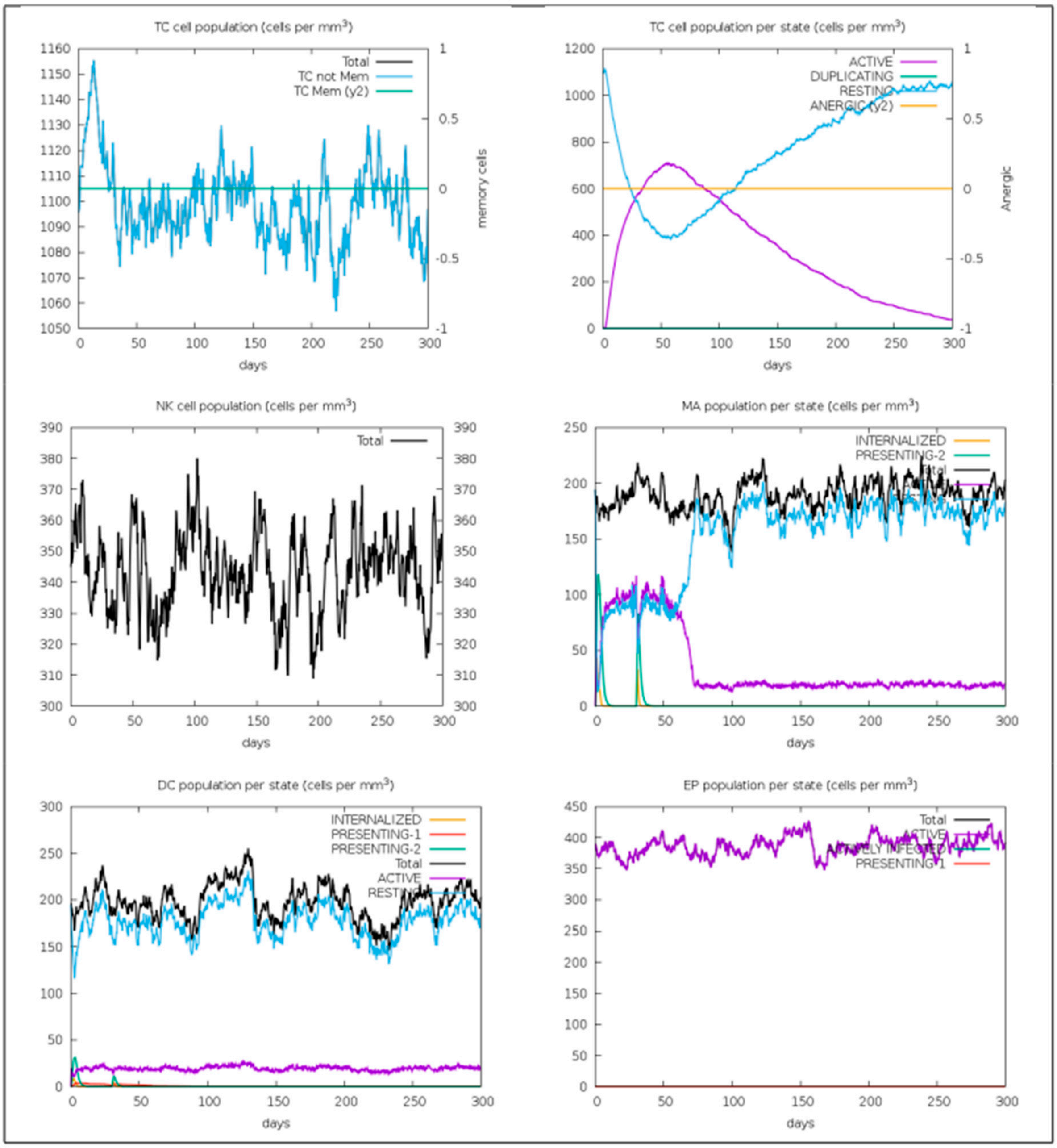

Effect of proposed MPXV-1-Beta on T-cell population and antigen-presenting cell

Figure S4. Results of Immune simulation after the second booster dose.

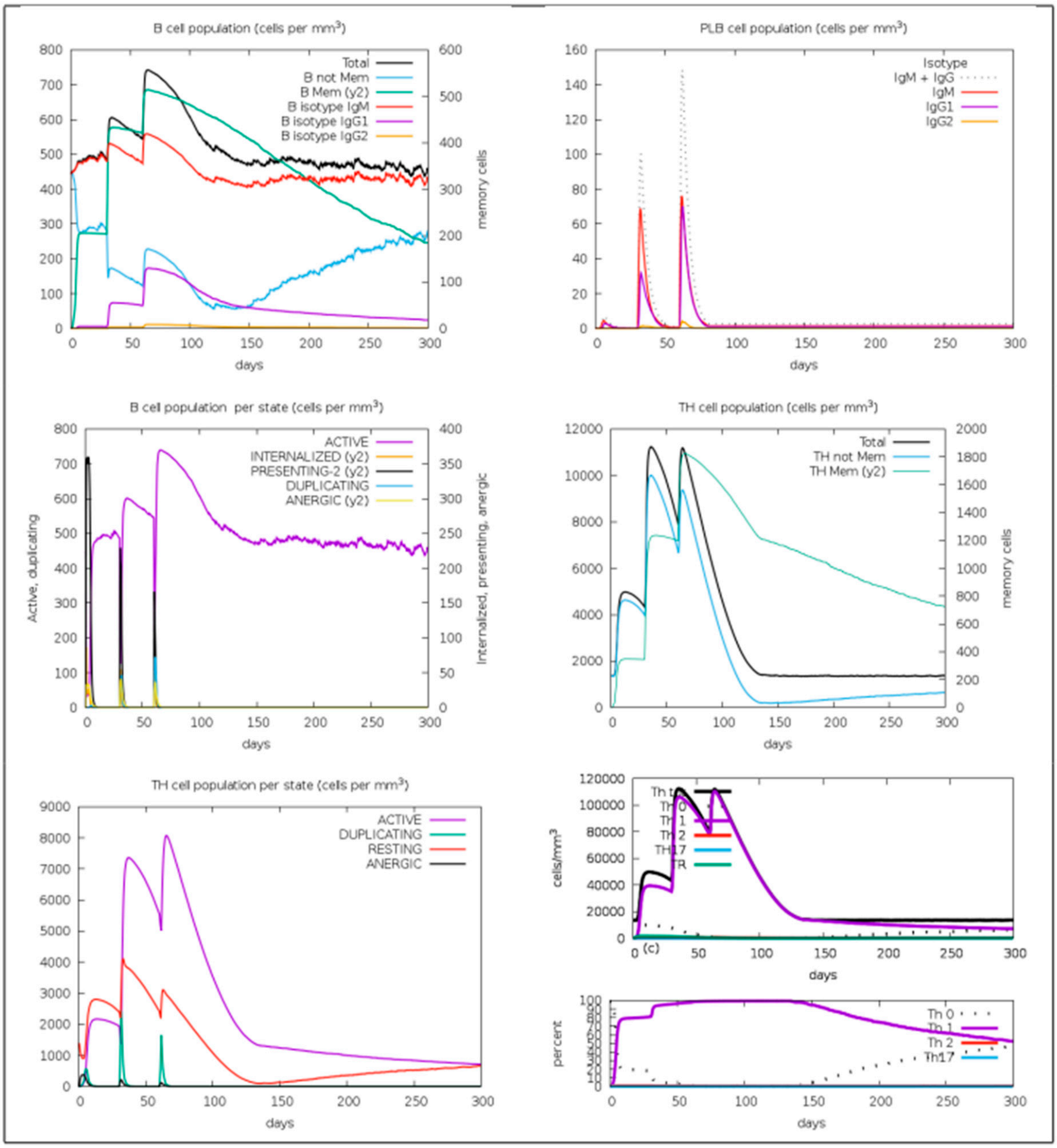

Effect of final dose of proposed MPXV-1-Beta formulation on B-cell population

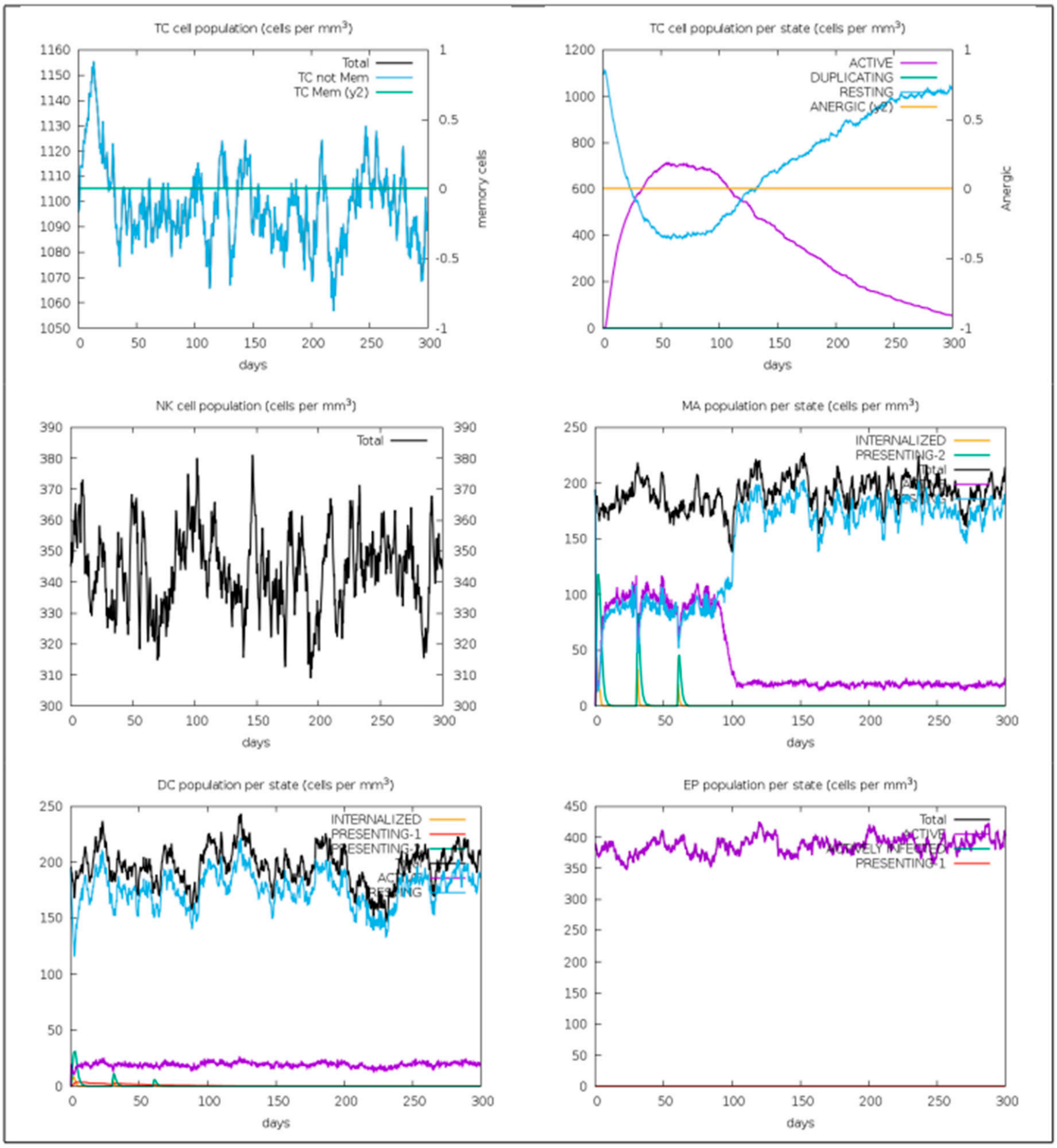

Effect of final dose of proposed on T-cell population and antigen-presenting cells

Figure S5. Effect of proposed MPXV-1-Beta formulation on populations of immune cells after final booster dose.
